# Canadian Society of Allergy and Clinical Immunology annual scientific meeting 2016

**DOI:** 10.1186/s13223-017-0192-y

**Published:** 2017-04-07

**Authors:** Mohammad A. Alsayegh, Hanan Alshamali, Mousa Khadada, Amanda Ciccolini, Anne K. Ellis, Diana Quint, William Powley, Laurie Lee, Yahya Fiteih, Shairaz Baksh, Harissios Vliagoftis, Sebastien K. Gerega, Brad Millson, Katia Charland, Stephane Barakat, Xichun Sun, Ricardo Jimenez, Susan Waserman, Mark J. FitzGerald, Jacques Hébert, Josiane Cognet-Sicé, Kevin E. Renahan, Saiful Huq, Rishma Chooniedass, Scott Sawyer, Hans Pasterkamp, Allan Becker, Steven G. Smith, Shiyuan Zhang, Kavisha Jayasundara, Claire Tacon, Alex Simidchiev, Gilbert Nadeau, Necdet Gunsoy, Hana Mullerova, Frank Albers, Young Woong Kim, Casey P. Shannon, Amrit Singh, Helen Neighbour, Mark Larché, Scott J. Tebbutt, Annika Klopp, Lorena Vehling, Allan B. Becker, Padmaja Subbarao, Piushkumar J. Mandhane, Stuart E. Turvey, Malcolm R. Sears, Meghan B. Azad, and the Canadian Healthy Infant Longitudinal Development Study Investigators, Keely Loewen, Barret Monchka, Salaheddin M. Mahmud, Geert ‘t Jong, Cristina Longo, Gillian Bartlett, Francine M. Ducharme, Tibor Schuster, Brenda MacGibbon, Tracie Barnett, Michelle L. North, Jeff Brook, Elizabeth Lee, Vanessa Omana, Jenny Thiele, Lisa M. Steacy, Greg Evans, Miriam Diamond, Gordon L. Sussman, Yann Amistani, Kathy Abiteboul, Mark W. Tenn, ChenXi Yang, Christopher Carlsten, Edward M. Conway, Douglas Mack, Yasmin Othman, Colin M. Barber, Chrystyna Kalicinsky, Andrea E. Burke, Mary Messieh, Parameswaran Nair, Chun T. Che, Lindsay Douglas, Joel Liem, Lucy Duan, Charlotte Miller, Pascale Dupuis, Lori A. Connors, Michael N. Fein, Joseph Shuster, Hani Hadi, Brooke Polk, Nikita Raje, Roxane Labrosse, Philippe Bégin, Louis Paradis, Anne Des Roches, Jonathan Lacombe-Barrios, Sanju Mishra, Gina Lacuesta, Meredith Chiasson, Babar Haroon, Kara Robertson, Thomas Issekutz, Desmond Leddin, Stephen Couban, Lori Connors, Adrienne Roos, Amin Kanani, Edmond S. Chan, Robert Schellenberg, Lana Rosenfield, Anna Cvetkovic, Kevin Woodward, Jaclyn Quirt, Wade T. A. Watson, Edson Castilho, Jennifer A. Sullivan, Beverley Temple, Donna Martin, Victoria E. Cook, Christopher Mills, Elodie Portales-Casamar, Lisa W. Fu, Alexander Ho, Jeffrey Zaltzman, Lucy Chen, Peter Vadas, Sofianne Gabrielli, Ann Clarke, Harley Eisman, Judy Morris, Lawrence Joseph, Sebastien LaVieille, Moshe Ben-Shoshan, François Graham, Charles Barnes, Jay Portnoy, Vincent Stagg, Elinor Simons, Diana Lefebvre, David Dai, Piushkumar Mandhane, Malcolm Sears, Herman Tam, F. Estelle R. Simons, Dhaifallah Alotaibi, Bassel Dawod, Matthew C. Tunis, Jean Marshall, Marylin Desjardins, Marianne Béland, Duncan Lejtenyi, Jean-Phillipe Drolet, Martine Lemire, Christos Tsoukas, Francisco J.D. Noya, Reza Alizadehfar, Christine T. McCusker, Bruce D. Mazer, Danay Maestre-Batlle, Evelyn Gunawan, Christopher F. Rider, Anette K. Bølling, Olga M. Pena, Daniel Suez, Isaac Melamed, Iftikhar Hussain, Mark Stein, Sudhir Gupta, Kenneth Paris, Sandor Fritsch, Christelle Bourgeois, Heinz Leibl, Barbara McCoy, Martin Noel, Leman Yel, Ori Scott, Brenda Reid, Adelle Atkinson, Vy Hong-Diep Kim, Chaim M. Roifman, Eyal Grunebaum, Eiman AlSelahi, Fernando Aleman, Amber Oberle, Mike Trus, Gordon Sussman, Amin S. Kanani, Olivier Chambenoi, Sima Chiva-Razavi, Savannah Grodecki, Nikhil Joshi, Peter Menikefs, David Holt, Teresa Pun, Damian Tworek, Raphael Hanna, Delia Heroux, Elli Rosenberg, Leah Stiemsma, Stuart Turvey, Judah Denburg, Christopher Mill, Timothy Teoh, Preeti Zimmer, Vishal Avinashi, Mihaela Paina, Ahmed A. Darwish Hassan, John Paul Oliveria, Chris Olesovsky, Gail Gauvreau, Linda Pedder, Paul K. Keith, Greg Plunkett, Michelle Bolner, Persia Pourshahnazari, Donald Stark, Kateryna Vostretsova, Andrew Moses, Andrew Wakeman, Alexander Singer, Thomas Gerstner, Elissa Abrams, Sara F. Johnson, Roberta L. Woodgate

**Affiliations:** 1 Allergy Division, Department of Medicine, Mubarak Alkabeer Hospital, Jabriya, Kuwait; 2 Respiratory Division, Department of Medicine, Medical Faculty, Kuwait University, Jabriya, Kuwait; 3 Department of Pediatrics, Queen’s University, Kingston, ON Canada; 4 Division of Allergy and Immunology, Department of Medicine, Queen’s University, Kingston, ON Canada; 5 Allergy Research Unit, Kingston General Hospital, Kingston, ON Canada; 6 Respiratory Therapeutic Area, GlaxoSmithKline, Stevenage, UK; 7 Pulmonary Research Group, Department of Medicine, University of Alberta, Edmonton, AB Canada; 8 Department of Pediatrics, University of Alberta, Edmonton, AB Canada; 9 IMS Brogan, Kirkland, QC Canada; 10 Teva Pharmaceutical Industries, Montreal, QC Canada; 11 Teva Pharmaceutical Industries, Frazer, PA USA; 12 McMaster University, Hamilton, ON Canada; 13 The Lung Centre, Vancouver, BC Canada; 14 Centre de recherche appliquée en allergie de Québec, Montreal, QC Canada; 15 Stallergenes Greer, Antony, France; 16 Stallergenes Greer, Cambridge, MA USA; 17 Department of Pediatrics and Child Health, University of Manitoba, Winnipeg, MB Canada; 18 Department of Pediatrics Emergency Medicine, Children’s Hospital, Winnipeg, MB Canada; 19 Children’s Hospital Research Institute of Manitoba, Winnipeg, MB Canada; 20 GlaxoSmithKline, Mississauga, ON Canada; 21 GlaxoSmithKline, Stockley Park, Uxbridge, UK; 22 GlaxoSmithKline, Research Triangle Park, NC USA; 23 Experimental Medicine, University of British Columbia, Vancouver, BC Canada; 24 James Hogg Research Centre for Heart Lung Innovation, St. Paul’s Hospital, Vancouver, BC Canada; 25 Prevention of Organ Failure (PROOF) Centre of Excellence, Vancouver, BC Canada; 26 Departments of Medicine and Biomedical and Molecular Science, Queen’s University, Kingston, ON Canada; 27 Department of Medicine, McMaster University, Hamilton, ON Canada; 28 Firestone Institute for Respiratory Health, McMaster University, Hamilton, ON Canada; 29 Division of Respiratory Medicine, Department of Medicine, University of British Columbia, Vancouver, BC Canada; 30 Department of Pediatrics and Child Health, University of Manitoba, Children’s Hospital Research Institute of Manitoba, Winnipeg, MB Canada; 31 Department of Pediatrics, Hospital for Sick Children, University of Toronto, Toronto, ON Canada; 32 Department of Pediatrics, Child and Family Research Institute, BC Children’s HospitalUniversity of British Columbia, Vancouver, BC Canada; 33 Max Rady College of Medicine, University of Manitoba, Winnipeg, MB Canada; 34 Vaccine and Drug Evaluation Centre, Department of Community Health Sciences, University of Manitoba, Winnipeg, MB Canada; 35 Centre for Health Care Innovation, Department of Community Health Sciences, University of Manitoba, Winnipeg, MB Canada; 36 Department of Family Medicine, McGill University, Montreal, QC Canada; 37 Centre de recherche du CHU Sainte-Justine, Montreal, QC Canada; 38 Department of Pediatrics, CHU Sainte-Justine, Montreal, QC Canada; 39 Department of Social and Preventive Medicine, Université de Montréal, Montreal, QC Canada; 40 Département de Mathématiques, Université du Québec à Montréal, Montreal, QC Canada; 41 Unité d’Épidémiologie et Biostatistiques, INRS-Institut Armand-Frappier, Laval, QC Canada; 42 Department of Biomedical and Molecular Science, Queen’s University, Kingston, ON Canada; 43 Department of Chemical Engineering and Applied Chemistry, University of Toronto, Toronto, ON Canada; 44 Southern Ontario Centre for Atmospheric Aerosol Research, University of Toronto, Toronto, ON Canada; 45 Air Quality Research Division, Environment Canada, Toronto, ON Canada; 46 Department of Earth Sciences, University of Toronto, Toronto, ON Canada; 47 Division of Allergy and Clinical Immunology, University of Toronto, Toronto, ON Canada; 48 Department of Biomedical and Molecular Sciences, Queen’s University, Kingston, ON Canada; 49 James Hogg Research Centre for Heart Lung Innovation, University of British Columbia, Vancouver, BC Canada; 50 Centre for Blood Research, University of British Columbia, Vancouver, BC Canada; 51 Department of Medicine, Division of Respiratory Medicine, University of British Columbia, Vancouver, BC Canada; 52 Pathology and Laboratory Medicine, University of British Columbia, Vancouver, BC Canada; 53 Department of Medicine, Mubarak Al-Kabeer Hospital, Jabriya, Kuwait; 54 Department of Pediatrics, McMaster University, Hamilton, ON Canada; 55 Department of Internal Medicine, Section of Clinical Immunology and Allergy, University of Manitoba, Winnipeg, MB Canada; 56 Division of Clinical Allergy and Immunology, Department of Medicine, McMaster University, Hamilton, ON Canada; 57 Division of Respirology, Department of Medicine, McMaster University, Hamilton, ON Canada; 58 Department of Pediatrics, University of Saskatchewan, Saskatoon, SK Canada; 59 Windsor Allergy Asthma Education Centre, Windsor, ON Canada; 60 Schulich School of Medicine and Dentistry, Western University, Windsor, ON Canada; 61 Allergy and Clinical Immunology, Department of Pediatrics, St. Joseph’s Health Centre, Toronto, ON Canada; 62 Department of Medicine, Dalhousie University, Halifax, NS Canada; 63 Allergy and Immunology, Dalhousie University, Halifax, NS Canada; 64 Department of Clinical Immunology and Allergy, McGill University, Montreal, QC H3G 1A4 Canada; 65 Division of Allergy and Immunology, Department of Pediatrics, Children’s Mercy HospitalUniversity of Missouri, Kansas City, MO USA; 66 Pediatric Allergy and Clinical Immunology, CHU Sainte-Justine, University of Montreal, Montreal, QC Canada; 67 Allergy and Clinical Immunology, CHUM, University of Montreal, Montreal, QC Canada; 68 Department of Medicine, Division of General Internal Medicine (Allergy and Immunology), Dalhousie University, Halifax, NS Canada; 69 Department of Medicine, Division of Respirology, Dalhousie University, Halifax, NS Canada; 70 Department of Critical Care and Department of Medicine, Division of General Internal Medicine, Dalhousie University, Halifax, NS Canada; 71 Department of Pediatrics, Dalhousie University, Halifax, NS Canada; 72 Division of Immunology, IWK Health Centre, Halifax, NS Canada; 73 Division of Gastroenterology, QEII Health Sciences Centre, Halifax, NS Canada; 74 Division of Hematology, QEII Health Sciences Centre, Halifax, NS Canada; 75 Halifax Allergy and Asthma Associates, Halifax, NS Canada; 76 Division of Allergy and Clinical Immunology, Department of Medicine, University of British Columbia, St Paul’s Hospital, Vancouver, BC Canada; 77 Division of Allergy and Immunology, Department of Pediatrics, Faculty of Medicine, University of British ColumbiaChildren’s Hospital, Vancouver, BC Canada; 78 Division of Clinical Immunology and Allergy, McMaster University, Hamilton, ON Canada; 79 Department of Internal Medicine, McMaster University, Hamilton, ON Canada; 80 Division of Infectious Diseases, McMaster University, Hamilton, ON Canada; 81 Division of Allergy, Department of Pediatrics, Dalhousie University, Halifax, NS Canada; 82 IWK Health Centre, Halifax, NS Canada; 83 College of Nursing, Faculty of Health Sciences, University of Manitoba, Winnipeg, MB Canada; 84 Division of Allergy and Immunology, Department of Pediatrics, University of British Columbia, BC Children’s Hospital, Vancouver, BC Canada; 85 Division of Allergy and Clinical Immunology, Department of Medicine, University of Toronto, Toronto, ON Canada; 86 Department of Anesthesia, University of Toronto, Toronto, ON Canada; 87 Division of Nephrology, Department of Medicine, University of Toronto, Toronto, ON Canada; 88 Division of Pediatric Allergy and Clinical Immunology, Department of Pediatrics, McGill University Health Center, Montreal, QC Canada; 89 Division of Rheumatology, Department of Medicine, University of Calgary, Calgary, AB Canada; 90 Department of Emergency Medicine, Montreal Children’s Hospital, McGill University Health Centre, Montreal, QC Canada; 91 Department of Emergency Medicine, Hôpital du Sacré-Coeur, Montreal, QC Canada; 92 Department of Epidemiology and Biostatistics, McGill University, Montreal, QC Canada; 93 Food Directorate, Health Canada, Ottawa, ON Canada; 94 Department of Allergy and Immunology, Centre Hospitalier de l’Université de Montréal Hôpital Notre-Dame, Montreal, QC Canada; 95 Department of Pediatrics, Allergy and Immunology Division, Centre Hospitalier Universitaire Sainte-Justine, Montreal, QC Canada; 96 Health Services and Outcomes Research, University of Missouri, Kansas City, MO USA; 97 Section of Allergy and Immunology, Department of Pediatrics and Child Health, University of Manitoba, Winnipeg, MB Canada; 98 Department of Pediatrics, British Columbia Children’s Hospital, Vancouver, BC Canada; 99 Department of Pediatrics, The Hospital for Sick Children, Toronto, ON Canada; 100 Division of Pediatric Respirology, Pulmonary and Asthma, Edmonton, AB Canada; 101 Pulmonary Research Group, Faculty of Medicine, University of Alberta, Edmonton, AB Canada; 102 Department of Pathology, Dalhousie University, Halifax, NS Canada; 103 Department of Microbiology and Immunology, Dalhousie University, Halifax, NS Canada; 104 Department of Pediatrics, McGill University, Montreal, QC Canada; 105 Meakins-Christie Laboratories, Research Institute of McGill University Health Centre, Montreal, QC Canada; 106 Department of Medicine, Université Laval, Quebec, QC Canada; 107 Department of Medicine, McGill University, Montreal, QC Canada; 108 Department of Respiratory Medicine, University of British Columbia, Vancouver, BC Canada; 109 Department of Air Pollution and Noise, Norwegian Institute of Public Health, Oslo, Norway; 110 Allergy, Asthma and Immunology Clinic, PA, Irving, TX USA; 111 IMMUNOe Health Centers, Centennial, CO USA; 112 Vital Prospects Clinical Research Institute, PC, Tulsa, OK USA; 113 Allergy Associates of the Palm Beaches, North Palm Beach, FL USA; 114 University of California at Irvine, Irvine, CA USA; 115 LSU Health Sciences Center, Children’s Hospital of New Orleans, New Orleans, LA USA; 116 Independent Consultant, Vienna, Austria; 117 Shire, Vienna, Austria; 118 Shire, Cambridge, MA USA; 119 Shire, Bannockburn, IL USA; 120 Department of Paediatrics, The Hospital for Sick Children, Toronto, ON Canada; 121 Division of Paediatric Immunology and Allergy, The Hospital for Sick Children, Toronto, ON Canada; 122 Division of Respirology and Department of Pathology and Molecular Medicine, McMaster University, Hamilton, ON Canada; 123 Division of Allergy and Clinical Immunology, St Michael’s Hospital, University of Toronto, Toronto, ON Canada; 124 Division of Allergy and Immunology, Department of MedicineUniversity of British Columbia, Vancouver, BC Canada; 125 Novartis Pharmaceuticals Canada Inc, Dorval, QC Canada; 126 Royal College of Surgeons, Dublin, Ireland; 127 Department of Medicine, University of Manitoba, Winnipeg, MB Canada; 128 Department of Anesthesia, St Joseph’s Health Centre, Toronto, ON Canada; 129 Community Allergist, Toronto, ON Canada; 130 Division of Allergy and Clinical Immunology, Department of Medicine, McMaster University, Hamilton, ON Canada; 131 Department of Microbiology and Immunology, University of British Columbia, Vancouver, BC Canada; 132 Department of Pediatrics, University of British Columbia, Vancouver, BC Canada; 133 Division of Allergy and Immunology, Department of Pediatrics, University of British Columbia, Vancouver, BC Canada; 134 Division of Gastroenterology, Hepatology and Nutrition, Department of Pediatrics, University of British Columbia, Vancouver, BC Canada; 135 Faculty of Medicine, University of British Columbia, Vancouver, BC Canada; 136 ALKInc., Round Rock, TX USA; 137 Department of Medicine, Division of Allergy and Clinical Immunology, University of British Columbia, Vancouver, BC Canada; 138 University College Dublin, Dublin, Ireland; 139 Department of Family Medicine, University of Manitoba, Winnipeg, MB Canada; 140 College of Nursing, Rady Faculty of Health Sciences, University of Manitoba, Winnipeg, MB Canada

## Allergic Rhinitis/Asthma

### A2 Common causes of allergic rhinitis for Kuwaiti residents

#### Mohammad A. Alsayegh^1^, Hanan Alshamali^1^, Mousa Khadada^1,2^

##### ^1^Allergy Division, Department of Medicine, Mubarak Alkabeer Hospital, Jabriya, Kuwait, ^2^Respiratory Division, Department of Medicine, Medical Faculty, Kuwait University, Jabriya, Kuwait

###### **Correspondence:** Mohammad A. Alsayegh

*Allergy, Asthma & Clinical Immunology* 2017, **13(Suppl 1)**:A2

**Background**: Multiple animal antigens, spores and pollens were collected and identified from the Kuwaiti atmosphere. The role for these antigens in mediating allergic rhinitis for Kuwaiti residents needs to be evaluated.

**Objective**: To investigate the causes (both indoors and outdoors) of allergic rhinitis for Kuwaiti residents.

**Method**: This is a retrospective study of all positive skin tests that we obtained in our Allergy clinic in Mubarak Alkabeer Hospital in Kuwait, during the period between May 2013 and December 2015, from patients who presented with allergic rhinitis symptoms and/or signs. They underwent skin prick tests to a battery of common allergens (german cockroach, cat dander, dog dander, house dustmites mix, cladosporium, aspergillus mix, penicillium mix, alternaria, grass pollens mix, Russian thistle pollens, mugwort pollens, rough pigweed pollens, sorrel pollens, compositae pollens, olive pollens, and date palm pollens). A wheal of ≥3 mm was considered a positive skin test.

**Results**: A total of 177 patients with rhinitis (90 females and 87 males) had positive test results to at least one allergen and were considered allergic. 77.9% of the patients had positive results to Russian thistle pollens, 39.9% to cat dander, 29.9% to grass pollens mix, 22.6% to compositae pollens, 22.6% to mugwort pollens, 22% to house dust mites mix, 21.4% to olive pollens, 20.9% to German cockroach, 20.3% to dog dander, 18.1% to rough pigweed pollens, 15.8% to date palm pollens, and 12.4% to sorrel pollens, 14.7% to penicillium, 10.7% to cladosporium, 10.7% to aspergillus mix, and 4% to alternaria.

**Conclusion**: Russian Thistle pollen is the commonest sensitization for Kuwaiti residents with allergic rhinitis.

### A3 Nature and prevalence of environmental allergies in the Kingston, Ontario region

#### Amanda Ciccolini^1^, Anne K. Ellis^2^

##### ^1^Department of Pediatrics, Queen’s University, Kingston, ON, Canada, ^2^Division of Allergy and Immunology, Department of Medicine, Queen’s University, Kingston, ON, Canada

###### **Correspondence:** Amanda Ciccolini

*Allergy, Asthma & Clinical Immunology* 2017, **13(Suppl 1)**:A3

**Background**: Environmental allergies affect many individuals of all ages. There are several aeroallergens that can trigger allergic reactions, namely allergic rhinitis and allergic asthma. In this study, we aimed to determine the prevalence of various environmental allergies in the Kingston, Ontario region.

**Methods**: A chart review of skin prick test (SPT) results was completed on all patients in the practice of an academic Allergist affiliated with Queen’s University. Patients who demonstrated positive SPT (defined as ≥3 mm than the negative control) to one or more allergens were included, and their age, gender and specific positive tests were recorded. Allergens evaluated included dust mites (*D. pteronyssinus* and *D. farinae*), dog dander, cat epithelium, tree mixes, birch pollen, other trees, grass mixes, ragweed mixes, short ragweed, other weeds, cockroach and numerous moulds. Of all patients reviewed, 1161 had positive SPT results to one or more allergens. Data analysis was completed with SPSS.

**Results**: Dust mite was the most prevalent allergen (62.6%). The second and third most common were ragweed (52.6%) and cats (51.6%), respectively. The prevalence of other allergens, in order of decreasing frequency, were grass (49.7%), trees (43.1%), birch (34.8%), short ragweed (30.8%), molds (29.7%), other trees (25.6%), dog (23.9%), cockroach (23.4%), other weeds (16.6%), with various molds being the least common (5.5%). There was a higher prevalence of environmental allergies in females (63.4%) compared to males (36.6%) both overall and for each specific allergen.

**Conclusion**: Environmental allergies are common. This study is the first to identify the prevalence of various environmental allergies in the Kingston, Ontario region, in line with anticipated results.

### A4 Evaluation of nasal responsiveness to allergen after treatment with intranasal TLR7 agonist, GSK2245035: a 1-year follow-up study

#### Anne K. Ellis^1,2^, Diana Quint^3^, William Powley^3^, Laurie Lee^3^

##### ^1^Allergy Research Unit, Kingston General Hospital, Kingston, ON, Canada, ^2^Department of Medicine, Queen’s University, Kingston, ON, Canada, ^3^Respiratory Therapeutic Area, GlaxoSmithKline, Stevenage, UK

###### **Correspondence:** Anne K. Ellis

*Allergy, Asthma & Clinical Immunology* 2017, **13(Suppl 1)**:A4

**Background**: Weekly treatment of seasonal allergic rhinitis patients during pollen seasons with intranasal GSK2245035 (20 or 80 ng), a toll-like receptor 7 (TLR7) agonist, reduced total nasal symptom score (TNSS) on nasal allergen challenge (NAC) 1 and 3 weeks post 8 weeks’ treatment (NCT01788813). The duration of effect of GSK2245035 on TNSS is unknown.

**Methods**: 16 of 21 (6 from the placebo arm; 10 from the 20 ng arm) participants who completed NCT01788813 in 2014 enrolled in this single centre, single period study (NCT02446613). No further study treatment was administered. A NAC was conducted with the allergen previously studied, approximately 1 year after treatment with GSK2245035 (follow up visit 3 [FUV3]). The TNSS was measured and nasal fluids collected for measurement of inflammatory biomarkers before and up to 6 h after NAC. Participants and staff remained blinded to the treatment they previously received. The repeated measures statistical modelling approaches (TNSS and biomarkers) were modified to include the additional 1 year timepoint and to remove the 80 ng data points. Derived TNSS endpoints (e.g. Weighted Mean 15 min) were formally modelled (individual TNSS components were summarised with simple summary statistics at each collection timepoint).

**Results**: At 15 min post NAC, the posterior probability (PP) for change from baseline in TNSS ≤ 0 was 0.107 for GSK2245035 20 ng compared with placebo. The probabilities for an increase in allergen specific IgA or a decrease in ECP, IL-5 and IL-16 in nasal fluids were each >0.7 at FUV3.

**Conclusions**: There was no evidence for an effect of i.n. GSK2245035 on TNSS post NAC 1 year after completing an 8 week study. A trend for changes in selected nasal allergic biomarkers was noted. The study conclusions are limited by the small sample size and duration of time between follow up visits.

**Funding**: GSK (NCT01788813 & NCT02446613).

### A5 Role of receptor interacting protein 2 in allergic airway inflammation

#### Yahya Fiteih^1^, Shairaz Baksh^2^, Harissios Vliagoftis^1^

##### ^1^Pulmonary Research Group, Department of Medicine, University of Alberta, Edmonton, AB, Canada, ^2^Department of Pediatrics, University of Alberta, Edmonton, AB, Canada

###### **Correspondence:** Yahya Fiteih

*Allergy, Asthma & Clinical Immunology* 2017, **13(Suppl 1)**:A5

**Background**: Persistent NF-κB activation has been associated with allergic airway inflammation in asthma. Receptor interacting protein 2 (RIP2) is a serine/threonine kinase that has been implicated in NF-κB activation. RIP2 polymorphism has been associated with severe childhood asthma. Furthermore, RIP2 gene silencing attenuated airway inflammation and airway hyper-responsiveness in ovalbumin-induced mouse asthma model. However, the mechanism of RIP2-mediated airway inflammation is not fully understood. To further investigate the role of RIP2 in asthma we will explore the effect of inhibiting RIP2 in mouse model of asthma using a new selective inhibitor.

**Methods**: Male Balb/c mice (6–8 weeks old) were sensitized twice with ovalbumin (10 µg) + aluminium hydroxide (2 mg) via intraperitoneal (i.p) injection and then challenged twice with ovalbumin (50 µg) intranasally. A new selective RIP2 inhibitor (RIP2 inhibitor-1) designed by Dr. Baksh, was administrated once daily for 3 days during challenge (1 µg/g body weight) via i.p injection. Mice were euthanized 24 h after the final challenge and bronchoalveolar lavage (BAL) fluid was collected. Airway inflammation was assessed by determining total and differential cell counts in BAL fluid. Lung tissues were collected for cytokine and chemokine analysis. Other mice were evaluated for airway hyper-responsiveness 24 h after the final challenge using Flexivent (SCIREQ).

**Results**: There was significant decrease in the total number of cells and in the percentage of eosinophils in BAL fluid from mice treated with ovalbumin + RIP2 inhibitor-1 when compared to mice treated with ovalbumin only. RIP2 inhibitor-1 decreased the total number of cells in BAL fluid by 50.1 ± 17.33% and decreased the percentage of eosinophils in BAL fluid by 33.34 ± 5.05%. n = 3 (4 mice per group).

**Conclusion**: RIP2 inhibtor-1 attenuated airway inflammation in ovalbumin-induced experimental asthma. RIP2 inhibition may be a novel therapeutic approach for the treatment of asthma.

### A6 Characteristics of patients with mild to severe asthma in Canada

#### Sebastien K. Gerega^1^, Brad Millson^1^, Katia Charland^1^, Stephane Barakat^2^, Xichun Sun^3^, Ricardo, Jimenez^2^, Susan Waserman^4^, Mark J. FitzGerald^5^

##### ^1^IMS Brogan, Kirkland, QC, Canada, ^2^Teva Pharmaceutical Industries, Montreal, QC, Canada, ^3^Teva Pharmaceutical Industries, Frazer, PA, USA, ^4^McMaster University, Hamilton, ON, Canada, ^5^The Lung Centre, Vancouver, BC, Canada

###### **Correspondence:** Sebastien K. Gerega

*Allergy, Asthma & Clinical Immunology* 2017, **13(Suppl 1)**:A6

**Background**: This retrospective longitudinal study used primary care based electronic medical records (EMR) to characterize asthma patients presenting to outpatient clinics in Ontario, Canada. The study evaluated and compared demographics, eosinophil levels (EOS), markers of control, and risk of comorbidities between severe and mild/moderate asthma patients as classified using the Global Initiative for Asthma (GINA) guidelines.

**Methods**: Patients were classified into one of the 5 GINA steps (steps 1–3 defined as mild/moderate, 4–5 as severe). Uncontrolled asthma was assessed based on the frequent use of SABA, use of oral corticosteroids, pulmonary function, hospitalization due to asthma, and/or an unscheduled physician visit for asthma. Differences in demographics were assessed using univariate tests (e.g. Wilcoxon Rank Sum, Mantel–Haenszel), and Poisson regression with robust error variance estimation to assess the relative risk of comorbidities while controlling for confounders.

**Results**: Of the 5,049 eligible asthma patients, 63.2% were mild/moderate and 36.8% were severe. Severe patients were older (median age 37.0 severe, 28.0 mild/moderate; p < 0.001) and more likely to have a history of smoking (29.8% severe, 22.5% mild/moderate; p < 0.001). Approximately 40% of patients had EOS recorded in the EMR, with severe patients more likely to have elevated EOS (≥400 cells/µL) (18.5% severe, 13.9% mild/moderate; p = 0.007). Less than 1% of patients had serum IgE levels on record. Uncontrolled asthma during the 1-year follow-up was more common in severe patients (34.5% severe, 22.0% mild/moderate; p < 0.001). Controlling for age and sex, there was a 25% increased risk of depression and a 63% increased risk of rhinosinusitis in the severe relative to mild/moderate patients (relative risk [RR] 1.25, 95% confidence interval [CI] 1.02, 1.55; RR 1.63; 95% CI 1.30, 2.03).

**Conclusion**: Severe asthma patients were more likely to have elevated EOS and uncontrolled asthma. They were also older, more likely to have a smoking history, and at greater risk for depression and rhinosinusitis.

### A7 Efficacy and safety of 300IR 5-grass pollen sublingual tablet in polysensitized subjects with grass pollen-induced allergic rhinoconjunctivitis

#### Jacques Hébert^1^, Josiane Cognet-Sicé^2^, Kevin E. Renahan^3^

##### ^1^Centre de recherche appliquée en allergie de Québec, Montreal, Québec, Canada, ^2^Stallergenes Greer, Antony, France, ^3^Stallergenes Greer, Cambridge, MA, USA

###### **Correspondence:** Jacques Hébert

*Allergy, Asthma & Clinical Immunology* 2017, **13(Suppl 1)**:A7

**Background**: The efficacy and safety profile of 300IR 5-grass pollen sublingual tablet administered daily pre-and co-seasonally has been demonstrated in subjects with grass pollen-induced allergic rhinoconjunctivitis (ARC). Using data from 7 clinical trials (one pediatric) of the development program, we present the results in 1388 polysensitized subjects.

**Methods**: At enrollment, subjects with medically confirmed grass pollen-induced ARC underwent skin testing to 5-grass mix or timothy and a panel of geographically relevant aeroallergens. Those testing positive to 5 grass/timothy and at least one other allergen were considered polysensitized. The Combined Score (scale 0–3) equally weighing symptom and rescue medication scores was analyzed using an ANCOVA for the single or first pollen period of 4 studies. Adverse events (AEs) pooled from all studies were analyzed descriptively.

**Results**: 1201 (64%) of 1878 adults (300IR = 663, placebo = 538) and 187 (60%) of 312 pediatric subjects (300IR = 92, placebo = 95) were polysensitized. Significant differences from placebo in the mean Combined Score were observed in both actively treated adult (−0.14, relative difference: −26.2%) and pediatric (−0.24, relative difference: −36.3%) subsets. As per overall population, application-site reactions such as oral pruritus (adults: 24.9%, pediatrics: 19.6%), throat irritation (adults: 20.8%, pediatrics: 13.0%) and mouth edema (adults: 9.0%, pediatrics: 9.8%) were the most commonly reported AEs. Three serious drug-related AEs occurred in actively treated adults: hypersensitivity (one polysensitized subject), angioedema and diarrhea (two monosensitized subjects) and resolved.

**Conclusion**: 300IR 5-grass pollen sublingual tablet was effective in subjects with multiple sensitizations, whether adults or children. The safety profile in both polysensitized subpopulations was consistent with that of overall population.

**Trial registration**: Clinicaltrials.gov registration numbers: NCT00367640, NCT00409409, NCT00418379, NCT00619827, NCT00803244, NCT00955825

### A8 Respiratory analysis of healthy and ‘wheezy’ sounds from children’s emergency

#### Saiful Huq^1,3^, Rishma Chooniedass^1,3^, Scott Sawyer^1,2^, Hans Pasterkamp^3^, Allan Becker^1,3^

##### ^1^Department of Pediatrics and Child Health, University of Manitoba, Winnipeg, MB, Canada, ^2^Department of Pediatrics Emergency Medicine, Children’s Hospital, Winnipeg, MB, Canada, ^3^Children’s Hospital Research Institute of Manitoba, Winnipeg, MB, Canada

###### **Correspondence:** Saiful Huq

*Allergy, Asthma & Clinical Immunology* 2017, **13(Suppl 1)**:A8

**Background**: Asthma is the most common and earliest chronic, complex disease to present in childhood. Almost 50% of children will wheeze in preschool years, but by school age only a minority have “true” asthma. The International Study of Allergy and Asthma in Childhood (ISAAC) showed parental reporting of wheeze decreased by 40% after they were shown a video of a wheezing child. There are currently no standard tests to help define asthma in the preschool child.

**Methods**: We recruited preschool children from the Winnipeg Children’s Hospital Emergency Department with wheezing and controls with non-respiratory illness. We captured 20–30 s of audiovisual recordings and the power spectra were analyzed using R.A.L.E, a program developed by Dr. Pasterkamp for breath sound analysis.

**Results**: Recordings have been captured from 40 children with wheezing syndrome and from 8 controls (mean age: 6 ± 2.1 years). We processed breath sound analysis on recordings from ten participants (mean age: 5 ± 2.4 years); five who presented with ‘breathing difficulties’ and five age and sex matched controls. There were differences between the power spectra of healthy and abnormal breath sounds: Children with breathing difficulties had wider power spectra in the mid-frequencies compared to children with healthy breath sounds. These same breath sounds had indications of resonant frequencies that were not as prominent in controls.

**Conclusion**: In this preliminary study, there are notable differences between breath sounds of children with wheezing syndrome and controls, as defined by breath sound analysis. We continue to enrol controls for a more complete comparison. Our goal is to better define breath sounds of children with wheezing/asthma compared to those with isolated lower respiratory infections. Detecting breathing difficulties early in life may help better diagnose asthma in preschool children and, with appropriate therapy, prevent development of chronic, irreversible airway changes.

### A9 Identification and description of severe asthma patients in a cross-sectional study (IDEAL): Canadian subject results

#### Steven G. Smith^1^, Shiyuan Zhang^1^, Kavisha Jayasundara^1^, Claire Tacon^1^, Alex Simidchiev^1^, Gilbert Nadeau^1^, Necdet Gunsoy^2^, Hana Mullerova^2^, Frank Albers^3^

##### ^1^GlaxoSmithKline, Mississauga, ON, Canada, ^2^GlaxoSmithKline, Stockley Park, Uxbridge, UK, ^3^GlaxoSmithKline, Research Triangle Park, NC, USA

###### **Correspondence:** Kavisha Jayasundara

*Allergy, Asthma & Clinical Immunology* 2017, **13(Suppl 1)**:A9

**Background**: The IDEAL study described the proportion of severe asthma patients eligible for anti IL-5 and/or anti-IgE directed treatments according to clinical trial or licensed indications. This study presents the results of the Canadian subjects to assess the local generalizability of the results compared to the overall cohort.

**Methods**: IDEAL was a cross-sectional, single-visit, non-drug interventional study conducted in 6 countries. It included subjects aged ≥12 years with severe asthma defined according to the ATS/ERS guidelines by treatment with high-dose ICS plus an additional controller(s) for ≥12 months; this post hoc analysis presents result for the Canadian cohort. Assessments included blood sample, spirometry, and symptom/burden of illness questionnaires. Mepolizumab, omalizumab and reslizumab treatment eligibilities were based on licensed indications or on clinical trial when indications were not available.

**Results**: 190 Canadian subjects were enrolled, of which 166 met analysis criteria. After exclusion of subjects currently treated with omalizumab, 88 subjects were included (mean age = 52.8 years [SD 15.95]; 49% female). 15 subjects (17.0% [95% Exact CI 9.9, 26.6]) were eligible for mepolizumab and 15 (17.0% [9.9, 26.6]) were eligible for omalizumab by Canadian label criteria, whereas 6 subjects (6.8% [2.5, 14.3]) were eligible for reslizumab. Among the 15 mepolizumab eligible subjects, 3 (20.0% [4.3, 48.1]) were also eligible for omalizumab and 2 (13.3% [1.7, 40.5]) for reslizumab.

**Conclusions**: In this Canada-based severe asthma population defined by high-dose ICS use plus additional controller(s) not currently taking omalizumab, a similar proportions of patients (~17%) were either mepolizumab-eligible (i.e., uncontrolled with eosinophilic inflammation) or omalizumab-eligible (i.e., with an allergic phenotype). In those mepolizumab eligible subjects, ~20% may be eligible for omalizumab, confirming the limited treatment options in this defined uncontrolled severe asthma population with an eosinophilic phenotype. These subgroup results were in general consistent with those for the overall IDEAL cohort

Funded by GSK; 201722.

### A10 Investigating systemic immune responses in the pathophysiology of allergic rhinitis under the nasal allergen challenge model

#### Young Woong Kim^1,2,3^, Casey P. Shannon^3^, Amrit Singh^1,2,3^, Anne K. Ellis^4,5^, Helen Neighbour^6,7^, Mark Larché^6,7^, Scott J. Tebbutt^1,2,3,8^

##### ^1^Experimental Medicine, University of British Columbia, Vancouver, BC, Canada, ^2^James Hogg Research Centre for Heart Lung Innovation, St. Paul’s Hospital, Vancouver, BC, Canada, ^3^Prevention of Organ Failure (PROOF) Centre of Excellence, Vancouver, BC, Canada, ^4^Departments of Medicine and Biomedical and Molecular Science, Queen’s University, Kingston, ON, Canada, ^5^Allergy Research Unit, Kingston General Hospital, Kingston, ON, Canada, ^6^Department of Medicine, McMaster University, Hamilton, ON, Canada, ^7^Firestone Institute for Respiratory Health, McMaster University, Hamilton, ON, Canada, ^8^Department of Medicine (Division of Respiratory Medicine), University of British Columbia, Vancouver, BC, Canada

###### **Correspondence:** Young Woong Kim

*Allergy, Asthma & Clinical Immunology* 2017, **13(Suppl 1)**:A10

**Background**: Allergic rhinitis (AR) is an IgE-mediated inflammatory condition of the nasal mucosa induced after allergen exposure. The nasal allergen challenge (NAC) model allows investigation of AR pathophysiology with biological sampling. Our study was an investigation of transcriptome changes of the collected peripheral blood samples.

**Methods**: Nineteen participants with a clinical history of AR to cat underwent NAC with cat allergen. Clinical symptom changes were assessed by Total Nasal Symptom Score (TNSS) and Peak Nasal Inspiratory Flow (PNIF). Whole blood was collected at baseline and post NAC (1, 2 and 6 h). PAXgene blood lysates were applied to profile 770 immune genes using a NanoString nCounter assay. Gene expression data was normalized using nSolver (ver. 2.6). Statistical analyses of complete blood count (CBC) data and gene expression data were performed using the R statistical computing program {Packages: limma (ver. 3.26.8), Mfuzz (ver. 2.30.0), WGCNA (ver. 1.51)}.

**Results**: TNSS and PNIF had a strong negative correlation (Pearson’s correlation = −0.978) consistent with AR response. Neutrophils (1, 2 and 6 h) and lymphocytes (6 h) significantly (*p* value <0.05) increased, and neutrophil to lymphocyte ratio (NLR), an inflammation indicator, increased at 1 (p value = 0.067) and 2 (p value = 0.051) hours post NAC compared to baseline. 156 genes (≥1.2 fold-change over time course) clustered into 7 distinct gene expression groups using a time-series analysis and 11 gene modules associated with CBC data using a gene correlation network analysis. Four of 7 groups and three of 11 gene modules were associated with specific immune cell types and immune signaling pathways.

**Conclusions**: Systemic changes in blood cells and gene expression can be demonstrated during NAC. These changes may be of use to examine efficacy or a mechanism of action of AR treatments when combined with NAC.

### A11 Do infants fed expressed breast milk have an increased risk of developing childhood asthma compared to directly breastfed infants?

#### Annika Klopp^1^, Lorena Vehling^1^, Allan B. Becker^1^, Padmaja Subbarao^2^, Piushkumar J. Mandhane^3^, Stuart E. Turvey^4^, Malcolm R. Sears^5^, Meghan B. Azad^1^ and the CHILD Study Investigators^6^

##### ^1^Department of Pediatrics and Child Health, University of Manitoba, Children’s Hospital Research Institute of Manitoba, Winnipeg, MB, Canada, ^2^Department of Pediatrics, Hospital for Sick Children, University of Toronto, Toronto, ON, Canada, ^3^Department of Pediatrics, University of Alberta, Edmonton, AB, Canada, ^4^Department of Pediatrics, Child and Family Research Institute, BC Children’s Hospital, University of British Columbia, Vancouver, BC, Canada, ^5^Department of Medicine, McMaster University, Hamilton, ON, Canada, ^6^Canadian Healthy Infant Longitudinal Development Study

###### **Correspondence:** Annika Klopp

*Allergy, Asthma & Clinical Immunology* 2017, **13(Suppl 1)**:A11

**Background**: Epidemiologic evidence suggests that breastfeeding may reduce the risk of childhood asthma; however, very few studies have distinguished how breast milk is delivered (i.e. nursing at the breast or feeding of expressed breast milk).

**Methods**: The study included 2647 children in the Canadian Healthy Infant Longitudinal Development Study, a population based national birth cohort. The primary exposure was infant feeding mode, categorized at 3 and 6 months as: direct breast milk (DBM), indirect breast milk (IBM, any expressed breast milk in the previous two weeks), formula only, or mixed (DBM/IBM and formula). Data were collected prospectively by standardized caregiver questionnaires. The primary outcome was asthma diagnosis at 3 years of age, based on a standardized history and physical examination. Logistic regression was used to explore the relationship between modes of infant feeding and asthma while adjusting for relevant covariates.

**Results**: A total of 327 children were diagnosed with possible or probable asthma at 3 years of age (12% incidence). At 3 months, the distribution of feeding modes was 61% breast milk only (28% DBM, 33% any IBM), 14% formula only, 25% mixed. By 6 months, the distribution was 48% breast milk (27% DBM, 21% any IBM), 26% formula, 26% mixed. Compared to direct breastfeeding, any mode of infant feeding at 3 months that included IBM or formula was associated with an increased risk of asthma. These associations persisted after adjusting for ethnicity, maternal diagnosis of asthma, and other covariates (IBM: adjusted OR 1.61, CI 1.10–2.36; mixed feeding: aOR 1.69, CI 1.13–2.51; formula: aOR 2.07, CI 1.31–3.25). Modes of infant feeding at 6 months were not significantly associated with asthma.

**Conclusion**: Modes of infant feeding in the first 3 months are associated with asthma development. Direct breastfeeding is protective compared to formula feeding, while expressed breast milk and mixed feeding confer intermediate levels of protection.

### A12 Prenatal antibiotic exposure and childhood asthma: a population-based study

#### Keely Loewen^1,2^, Barret Monchka^3^, Salaheddin M. Mahmud^3,4^, Geert ‘t Jong^1,5^, Meghan B. Azad^1,5^

##### ^1^Children’s Hospital Research Institute of Manitoba, Winnipeg, MB, Canada, ^2^Max Rady College of Medicine, University of Manitoba, Winnipeg, MB, Canada, ^3^Vaccine and Drug Evaluation Centre, Department of Community Health Sciences, University of Manitoba, Winnipeg, MB, Canada, ^4^Centre for Health Care Innovation, Department of Community Health Sciences, University of Manitoba, Winnipeg, MB, Canada, ^5^Department of Pediatrics and Child Health, University of Manitoba, Winnipeg, MB, Canada

###### **Correspondence:** Keely Loewen

*Allergy, Asthma & Clinical Immunology* 2017, **13(Suppl 1)**:A12

**Background**: Antibiotic exposure during infancy has been linked to asthma, but few studies have evaluated prenatal antibiotic exposure. Antibiotics can have long-term health effects through induced dysbiosis of the gut microbiota and associated disruption of immune development. We investigated the association between maternal antibiotic use in pregnancy and childhood asthma, accounting for antibiotic type and trimester of exposure.

**Methods**: Using Manitoba’s health administrative data housed at the Manitoba Centre for Health Policy, we created a provincial birth cohort of mother–child dyads comprising all children born between 1996 and 2012. In utero antibiotic exposure was determined from maternal prescription records and child asthma was defined as: any hospitalization for asthma or ≥2 physician diagnoses of asthma or ≥2 prescriptions for asthma medications within a 1 year period after age 5. Associations were determined using Cox regression and expressed as hazard ratios (HR) and 95% confidence intervals (CI).

**Results**: In our study population of 213,661 children, 12% had asthma and 37% were exposed to antibiotics in utero. Prenatal exposure to antibiotics was positively associated with asthma (crude HR 1.30; 95% CI 1.27–1.33). This association persisted after controlling for sex, location of residence, gestational age, and number of siblings, but was attenuated after adjusting for healthcare utilization. Associations were similar regardless of the trimester of exposure. When classified by antibiotic type, associations were observed for penicillins, macrolides, and sulphonamides and trimethoprim, but not for other beta-lactams or tetracyclines. Ongoing analyses will account for additional covariates, number of antibiotic exposures and postnatal antibiotic use.

**Conclusion**: We found that antibiotic use during any trimester of pregnancy was associated with a modest increase in asthma risk among offspring. Healthcare utilization patterns appear to be an important confounder of this association.

### A13 Weight status and time to first exacerbation in children with asthma: is there a dose–response relationship?

#### Cristina Longo^1^, Gillian Bartlett^1^, Francine M. Ducharme^2,3,4^, Tibor Schuster^1^, Brenda MacGibbon^5^, Tracie Barnett^2,6^

##### ^1^Department of Family Medicine, McGill University, Montreal, QC, Canada, ^2^Centre de recherche du CHU Sainte-Justine, Montreal, QC, Canada, ^3^Department of Pediatrics, CHU Sainte-Justine, Montreal, QC, Canada, ^4^Department of Social and Preventive Medicine, Université de Montréal, Montreal, QC, Canada, ^5^Département de Mathématiques, Université du Québec à Montréal, Montreal, QC, Canada, ^6^Unité d’Épidémiologie et Biostatistiques, INRS-Institut Armand-Frappier, Laval, QC, Canada

###### **Correspondence:** Cristina Longo

*Allergy, Asthma & Clinical Immunology* 2017, **13(Suppl 1)**:A13

**Background**: Obesity has been linked with poor asthma control and decreased response to inhaled corticosteroids (ICS). However, other studies have refuted these claims.

**Objective**: To determine if weight status has a dose–response relationship with time to first exacerbation among children with asthma.

**Methods**: A historical cohort study was conducted from a clinical database that was linked to health and drug administrative databases. The cohort consisted of children aged 2–18 years with specialist-confirmed asthma, who consulted with the Asthma Centre (AC) of the Montreal Children’s Hospital between April 1st, 2000 and September 31st, 2007 in Quebec, Canada. We included children initiating higher-dose ICS monotherapy (MT) or ICS combination therapy (CT) at the clinic index date. We excluded children with: bronchopulmonary dysplasia, cystic fibrosis, or no public drug insurance coverage. The cohort end date was the date of first exacerbation, end of health insurance coverage, or end of 1-year follow-up, whichever occurred first. Sex-specific BMI for age percentiles were calculated using the WHO growth charts. Exacerbation was defined as an emergency department visit, hospital admission, or use of oral corticosteroids for asthma. A Cox model was used to assess the dose response between weight status, i.e. using BMI percentile categories, on the hazard of first exacerbation.

**Results**: 328 children were incident users of ICS MT (N = 231) or ICS CT (N = 97), with 234 (65.9%) events during follow-up. When compared to children in the 44th BMI percentile or less category, those belonging to the 45th to 64th BMI percentile class had a HR of 2.25 (95% CI 0.93–5.41), 65th to 84th BMI percentile had a HR of 4.35 (95% CI 0.99–19.01), and 85th BMI percentile or more had a HR of 8.36 (95% CI 1.09–64.08).

**Conclusions**: This study is the first to demonstrate the dose–response relationship between weight status and exacerbation-free time in pediatric asthma.

### A14 Kingston Allergy Birth Cohort (KABC); exposome characteristics and parentally reported respiratory outcomes to age 2

#### Michelle L. North^1,2,3,4^, Jeff Brook^4,5^, Elizabeth Lee^1^, Vanessa Omana^1^, Jenny Thiele^1,2^, Lisa M. Steacy^2,3^, Greg Evans^3,4^, Miriam Diamond^7^, Anne K. Ellis^1,2,3^

##### ^1^Department of Biomedical and Molecular Sciences, Queen’s University, Kingston, ON, Canada, ^2^Allergy Research Unit, Kingston General Hospital, Kingston, ON, Canada, ^3^Department of Chemical Engineering and Applied Chemistry, University of Toronto, Toronto, ON, Canada, ^4^Southern Ontario Centre for Atmospheric Aerosol Research, University of Toronto, Toronto, ON, Canada, ^5^Air Quality Research Division, Environment Canada, Toronto, ON, Canada, ^6^Division of Allergy and Immunology, Department of Medicine, Queen’s University, Kingston, ON, Canada, ^7^Department of Earth Sciences, University of Toronto, Toronto, ON, Canada

###### **Correspondence:** Michelle L. North

*Allergy, Asthma & Clinical Immunology* 2017, **13(Suppl 1)**:A14

**Background**: The Kingston Allergy Birth Cohort (KABC) was initiated to study the developmental origins of allergic disease. Kingston General Hospital serves a population with notable diversity in exposures relevant to the emerging concept of the exposome. The exposome concept organizes exposures across the life course into three domains; “general external” (population level; SES, rural/urban residency), “specific external” (individual level; cigarette smoke, breastfeeding, mold/dampness), and “internal” (respiratory health, gestational age). The objective of this study was to establish a profile of the KABC cohort using the exposome framework and examine parentally reported respiratory symptoms to age 2.

**Methods**: We determined the effects of the general external domain (urban/rural residence, SES) on specific external exposures by Fisher’s exact test or Chi squared test, and adjusted the resulting p values for multiple testing. Multivariate Cox proportional hazard models were used to determine associations between prenatal/postnatal factors and respiratory symptoms.

**Results**: Rural KABC participants were significantly less likely to report living within 100 m of major roads, construction and parking lots. However, they were significantly more likely to use wood for heat, have a furry pet inside the home, and to live near a farm. Lowest-tertile SES participants were significantly less likely to live near a farm and to use wood heating, but more likely to live in older homes, in proximity to major roads and parking lots, and to use air fresheners. Significant associations emerged between parental reports of wheeze/cough without a cold and SES, prenatal smoke exposure and mold/dampness. Respiratory symptoms were negatively associated with increasing gestational age and breastfeeding for at least the first six months of life.

**Conclusions**: The KABC is a unique cohort with diversity that can be leveraged for exposomics-based studies. This study demonstrated the impacts of all three domains of the exposome on the respiratory health of KABC children.

### A17 Safety over time of 5-grass pollen sublingual tablet in subjects with grass pollen-induced allergic rhinoconjunctivitis

#### Gordon L. Sussman^1^, Yann Amistani^2^, Kathy Abiteboul^2^

##### ^1^Division of Allergy and Clinical Immunology, University of Toronto, Toronto, ON, Canada, ^2^Stallergenes Greer, Antony, France

###### **Correspondence:** Gordon L. Sussman

*Allergy, Asthma & Clinical Immunology* 2017, **13(Suppl 1)**:A17

**Background**: Across the development program, treatment with 5-grass pollen sublingual tablet has a well established safety profile in subjects with grass pollen-induced allergic rhinoconjunctivitis (ARC). Here we present the safety data of the product over the pre- and co-seasonal treatment periods from a pooled analysis.

**Methods**: Subjects with medically confirmed grass pollen-induced ARC were enrolled in one of 8 double-blind studies. They received a 5-grass or placebo tablet daily 2 or 4 months pre- and co-seasonally (5 single season studies and over 3 years in a long-term study) or outside the pollen season (Phase I studies). Adverse events (AEs) from the first pre-co-seasonal treatment period (pool from all studies) and from 3 consecutive treatment periods (long-term study) were analyzed descriptively.

**Results**: The analysis included 2512 subjects (2200 adults and 312 pediatric subjects) of which 1514 received the active treatment and 998 the placebo. Drug-related AEs were reported by 58% of actively treated subjects and 20% of placebo-recipients. Events were mostly consistent with application-site reactions (e.g., oral pruritus, throat irritation, mouth edema). The majority of these events occurred within the first week of treatment initiation, most on the first day. In the long-term study, drug-related AE incidences in actively treated adults in the respective 2- and 4-month groups were 61 and 71% in Year 1, 54 and 59% in Year 2, and 40 and 45% in Year 3. Three serious treatment-related AEs (hypersensitivity, angioedema and diarrhea) were reported in Year 1 in adults, none in pediatrics.

**Conclusion**: The 5-grass pollen sublingual tablet showed a favorable safety profile in grass pollen allergic subjects. Adverse events were reported with decreasing incidences over consecutive seasons.

**Trial registration**: Clinicaltrials.gov registration numbers: NCT00367640, NCT00409409, NCT00418379, NCT00619827, NCT00803244, NCT00955825

### A20 Nasal and peripheral blood group 2 innate lymphoid cells (ILC2) levels in response to nasal allergen challenge in participants with allergic rhinitis

#### Mark W. Tenn^1^, Jenny Thiele^1,2^, Anne K. Ellis^1,2,3^

##### ^1^Department of Biomedical and Molecular Sciences, Queen’s University, Kingston, ON, Canada, ^2^Allergy Research Unit, Kingston General Hospital, Kingston, ON, Canada, ^3^Division of Allergy and Immunology, Department of Medicine, Queen’s University, Kingston, ON, Canada

###### **Correspondence:** Mark W. Tenn

*Allergy, Asthma & Clinical Immunology* 2017, **13(Suppl 1)**:A20

**Background**: Group 2 innate lymphoid cells (ILC2s) produce IL-5 and IL-13, drive Th2 inflammation, and are increased in the peripheral blood of individuals with allergic rhinitis (AR) after allergen challenge, and during pollen season. However, the identification of ILC2s in nasal samples has not been carefully examined. In participants with birch pollen-induced AR, we quantified the frequency of both nasal and peripheral ILC2s before and after a nasal allergen challenge (NAC).

**Methods**: 11 individuals with birch pollen-induced AR and 8 non-allergic individuals were recruited. Each participant underwent a NAC with birch pollen extract (ALK-Abello, Mississauga). Nasal lavage (NL) and peripheral blood were collected at baseline (before challenge) and 4 h post-challenge. NL samples were treated with dithiothreitol and peripheral blood mononuclear cells (PBMCs) were isolated and cryo-preserved. Fresh NL samples and thawed PBMCs were stained with fixable viability dye (PBMCs only), lineage (Lin) surface markers, CD45, CD294 (CRTH2), and CD127 (IL-7Rα) antibodies. Stained cells were acquired using a 4-colour Beckman Coulter Cytomics FACSCanto 500 flow cytometer and an 8-colour FACSAria III cell sorter. Data was analyzed using FlowJo software. GraphPad Prism 6 was used for statistical analysis.

**Results**: A trend was observed suggesting an increase in the frequency of both nasal ILC2s (defined as Lin CD45 + CRTH2 + IL-7Rα+) (p = 0.08) and nasal non-ILC2s (including ILC1s and ILC3s; defined as Lin CD45 + CRTH2-IL-7Rα +) (p = 0.06) in participants with birch pollen-induced AR following a NAC. This trend was absent for both cell subsets in peripheral blood.

**Conclusions**: In NL samples, we demonstrated a trend suggesting an increase in both nasal ILC2s and nasal non- ILC2s after allergen challenge. However, the frequency of peripheral ILC2s remained unchanged, contrasting with previous literature. This suggests that ILC2s and other ILC subsets (ILC1 and ILC3) may play a role locally in the nose in individuals with AR.

### A22 Discovery of biomarkers that can diagnose western red cedar asthma

#### ChenXi Yang^1,2,3,4^, Amrit Singh^1,2,4^, YoungWoong Kim^1,2,4^, Christopher Carlsten^1,5^, Edward M. Conway^3,6^, Scott J. Tebbutt^1,2,5^

##### ^1^James Hogg Research Centre for Heart Lung Innovation, University of British Columbia, Vancouver, BC, Canada, ^2^Prevention of Organ Failure (PROOF) Centre of Excellence, Vancouver, BC, Canada, ^3^Centre for Blood Research, University of British Columbia, Vancouver, BC, Canada, ^4^Experimental Medicine, University of British Columbia, Vancouver, BC, Canada, ^5^Department of Medicine, Division of Respiratory Medicine, University of British Columbia, Vancouver, BC, Canada, ^6^Pathology and Laboratory Medicine, University of British Columbia, Vancouver, BC, Canada

###### **Correspondence:** ChenXi Yang

*Allergy, Asthma & Clinical Immunology* 2017, **13(Suppl 1)**:A22

**Background**: Western red cedar asthma is the most common form of occupational asthma in the Pacific Northwest region of North America and affects 4.0–13.5% of the exposed population. It has been shown that individuals with western red cedar asthma are sensitive to plicatic acid (PA), which is a low molecular weight molecule found in the wood. In this study, peripheral whole blood gene expression was used as a source to identify biomarker panels which distinguish asthmatic individuals who are sensitive to PA (PA-positive) from asthmatic individuals who are not sensitive to PA (PA-negative).

**Methods**: Peripheral whole blood samples were collected using PAXgene Blood RNA tubes from 10 PA-positive subjects and 8 PA-negative subjects. Gene expression was profiled using a custom NanoString nCounter Elements assay. Sparse partial least squares discriminant analysis (sPLS-DA) was used to identify potential biomarker panels that distinguish the PA-positive subjects from the PA-negative subjects.

**Results**: Using sPLS-DA followed by a leave-one-out cross-validation, a 14-gene panel was shown to distinguish PA-positive subjects from PA-negative subjects at an AUC (area under the receiver operating characteristic [ROC] curve) of 0.82. The top 3 tissues related to these 14 genes are Myeloid CD33+ (FDR = 4.62 × 10^−7^), Monocyte CD14+ (FDR = 2.39 × 10^−5^) and γδ T cells (FDR = 5.02 × 10^−4^).

**Conclusions**: Peripheral blood can potentially be used as an easily obtainable biospecimen to diagnose western red cedar asthma. Further validation of the biomarker panel is required and is currently underway.

**Acknowledgements**: We would like to thank our research participants as well as Agnes Yuen and Darren Sutherland at the Chan-Yeung Center for Occupational and Environmental Respiratory Disease. This study was supported by WorkSafeBC, BC Lung Association, and Mitacs.

## Case Reports

### A23 Recurrent anaphylaxis successfully treated with colchicine

#### Mohammad A. Alsayegh^1^, Douglas Mack^2^, Yasmin Othman^1^

##### ^1^Department of Medicine, Mubarak Al-Kabeer Hospital, Jabriya, Kuwait, ^2^Department of Pediatrics, McMaster University, Hamilton, ON, Canada

###### **Correspondence:** Mohammad A. Alsayegh

*Allergy, Asthma & Clinical Immunology* 2017, **13(Suppl 1)**:A23

**Background:** Idiopathic anaphylaxis (IA) is a generalized systemic reaction to unknown triggering factors. We classify IA as frequent (six or more episodes in the past year) or infrequent (less than six episodes in the past year). The treatment of IA with frequent recurrence can be challenging. To our knowledge, we present the first case of successful treatment of IA with colchicine.

**Case presentation:** A 52-year-old female is known to have IA for 25 years, with frequent recurrence of symptoms, which required multiple courses of systemic corticosteroids in the past. She was admitted with an acute episode of IA that manifested by urticarial rash, facial angioedema, acute cough, abdominal discomfort, and hypotension. She is found to have active painful mouth ulcers on admission that she reports have been recurrent for more than 5 years. Other past medical history includes hypothyroidism for 25 years due to Hashimoto thyroiditis. Her regular medications included cetirizine, and thyroid hormone replacement. Thyroperoxidase antibody and total IgE were found to be elevated at 345 and 992 IU/mL respectively, and the total serum tryptase was normal. She was discharged on oral colchicine 0.5 mg twice daily, and oral prednisone 1 mg/kg once daily. She discontinued prednisone after 10 days. During a course of 42 months of follow up, she tolerated colchicine well, and reported good compliance with it. This patient has had no recurrence of mouth ulcers or anaphylaxis symptoms since commencing colchicine.

**Discussion:** Colchicine was prescribed for this patient to prevent recurrent mouth ulcers. The improvement of her recurrent IA was a positive side effect to the drug. More studies are needed to determine colchicine’s benefit for IA.

**Conclusion:** We have concluded that colchicine may be helpful in the treatment of IA.

**Consent to publish:** A written consent to publish was obtained from the patient to publish this data.

### A24 A novel combination of an IgE mediated adult onset food allergy and a suspected mast cell activation syndrome presenting as anaphylaxis

#### Colin M. Barber, Chrystyna Kalicinsky

##### Department of Internal Medicine, Section of Clinical Immunology and Allergy, University of Manitoba, Winnipeg, MB, Canada

###### **Correspondence:** Colin M. Barber

*Allergy, Asthma & Clinical Immunology* 2016, 12:46

This abstract is not included here as it has already been published [1].

**Reference**

1. Barber C and Kalicinsky C. A novel combination of an IgE mediated adult onset food allergy and a suspected mast cell activation syndrome presenting as anaphylaxis. Allergy, Asthma Clin Immunol 2016;12:46

### A25 Questioning the dogma of “physician-diagnosed asthma”

#### Andrea E. Burke^1^, Mary Messieh^1^, Parameswaran Nair^2^

##### ^1^Division of Clinical Allergy and Immunology, Department of Medicine, McMaster University, Hamilton, ON, Canada, ^2^Division of Respirology, Department of Medicine, McMaster University, Hamilton, ON, Canada

###### **Correspondence:** Andrea E. Burke

*Allergy, Asthma & Clinical Immunology* 2017, **13(Suppl 1)**:A25

**Background:** Epidemiological studies, clinical trials and clinical practice often rely on “physician-diagnosis” of asthma to draw conclusions from data and direct treatment. We present a case that illustrates the limitations of this dogma and the need to use objective clinical measurements to improve clinical outcomes.

**Case presentation:** A 31 year-old female non-smoker presented with asthma and recurrent bronchitis since childhood. Asthma had been diagnosed by specialists based on symptoms of intermittent wheezing, chest tightness and cough. These symptoms continued into adulthood with significant limitation in her work. She had been prescribed regular inhaled corticosteroids, long-acting beta-agonists and multiple courses of antibiotics and prednisone. A negative methacholine challenge suggested her symptoms were unlikely to represent variable airflow obstruction. Absence of eosinophils on quantitative sputum cytometry suggested that she did not have steroid-responsive airway disease. Persistent sputum neutrophilia and a high lipid index in sputum macrophages led to investigations identifying gastroesophageal reflux, a tracheal diverticulum and mild bronchomalacia as other possible contributors to her symptoms.

**Discussion:** We present a case of persistent respiratory symptoms treated as asthma, with inhaled and oral corticosteroids used over many years. In addition to other investigations, sputum analysis helped identify bacterial bronchitis and silent aspiration as a more likely explanation of her symptoms. Although difficult to prove, a small tracheal diverticulum may also have contributed. High doses of corticosteroids seem to have been not only unnecessary, but may have exacerbated infective bronchitis. Discontinuation of inhaled corticosteroids and beta-agonists, and the use of anti-reflux therapies now maintain her symptom-free.

**Conclusion:** This case illustrates the utility of clinical measurements of airway hyper-responsiveness and cellular bronchitis in the investigation and management of airway symptoms. They are particularly helpful in the evaluation of patients with complex airway diseases and the prevention of over-treatment of patients with mild airway disease.

**Consent to publish:** Consent to publish was obtained.

### A26 Cross reactive conundrums: what is the allergen?

#### Chun T. Che^1^, Lindsay Douglas^2^, Joel Liem^2,3^

##### ^1^Department of Pediatrics, University of Saskatchewan, Saskatoon, SK, Canada, ^2^Windsor Allergy Asthma Education Centre, Windsor, ON, Canada, ^3^Schulich School of Medicine and Dentistry, Western University, Windsor, ON, Canada

###### **Correspondence:** Chun T. Che

*Allergy, Asthma & Clinical Immunology* 2017, **13(Suppl 1)**:A26

**Background:** An increasingly encountered scenario is a peanut or cashew allergic patient reacting to a protein botanically related to peanut or cashew due to the possible cross reactivity between related proteins.

**Case presentations:** Case 1: 14 year old male with peanut allergy presented with pruritus and tingling lips within minutes of eating curry for the first time. The curry contained no proteins he was known to be allergic to. On repeat exposure, pruritus, hives, shortness of breath and nausea developed. In both cases, symptoms resolved with epinephrine. Skin prick testing (SPT) to 11 common curry components revealed a strong positive reaction to fenugreek. Case 2: 14 year old female with known food dependent exercise induced anaphylaxis and cashew allergy initially developed abdominal discomfort after eating Fattoush salad. Pruritus, cough and eyelid angioedema developed a few minutes into playing basketball. Symptoms resolved with epinephrine and oral glucocorticoids. On repeat exposure, abdominal discomfort and hives developed which resolved without medication. SPT revealed a strong positive reaction to sumac and hummus.

**Discussion:** Fenugreek, a spice used in curry, is from the same botanical family (Fabaceae) as peanuts. Case 1 is the first Canadian report of fenugreek allergy in a peanut allergic individual likely due to peanut- fenugreek cross reactivity.

Sumac, a main ingredient in Fattoush salad, is from the same botanical family (Anacardiaceae) as cashews. Case 2 is the first report of sumac allergy in a cashew allergic individual likely due to cashew-sumac cross reactivity. She has a known peanut allergy and has eaten hummus since this time without a reaction.

**Conclusion:** Although previously considered insignificant, cross reactivities seem to be becoming more important. We believe physicians should consider discussing potential cross reactivities with peanut or cashew allergic patients.

**Consent to publish:** Consent to publish their clinical details was obtained from both patients’ parents.

### A28 Serious allergic reaction with the fifth dose of sublingual immunotherapy for grass-pollen allergy

#### Lucy Duan^1^, Charlotte Miller^2^

##### ^1^Department of Pediatrics, Hospital for Sick Children, University of Toronto, Toronto, ON, Canada, ^2^Allergy and Clinical Immunology, Department of Pediatrics, St. Joseph’s Health Centre, Toronto, ON, Canada

###### **Correspondence:** Lucy Duan

*Allergy, Asthma & Clinical Immunology* 2017, **13(Suppl 1)**:A28

**Background:** Allergic rhinitis and conjunctivitis to grass-pollen is associated with significant morbidity and decreased quality of life in patients affected. Sublingual immunotherapy (SLIT) is a safe and effective treatment for this condition and reduces symptoms and the need for pharmacotherapy. GRASTEK (Timothy Grass Pollen Allergen Extract) is a sublingual immunotherapy approved for use in North America. Treatment related adverse events (TRAE) have been reported however severe reactions are rare.

**Case presentation:** A 17-year-old-female with a history of grass-pollen induced rhinoconjunctivitis was started on treatment with GRASTEK in March 2016. Her first dose was administered by her University Health Centre and an Epipen was prescribed based on the product warnings/precautions. She tolerated the first four doses of GRASTEK with no adverse events. About five minutes after taking the fifth dose, she developed oral pruritus and oropharyngeal pain followed shortly by dysphagia, dysphonia and feeling that she couldn’t breathe. There was no cough, skin, gastrointestinal or circulatory symptoms. She administered the Epipen herself, with complete symptom resolution. She has no history of asthma, food or medication allergies.

**Discussion:** Transient local reactions including oral itching and tongue swelling are a known occurrence with commencement of GRASTEK, but serious local and systemic reactions have rarely been reported. In the Phase II and III development of the 75,000-SQ timothy grass-pollen tablet, eight patients reported serious local and systemic allergic symptoms and four were treated with epinephrine. In all four cases, reactions occurred on the first day of treatment. Similarly, in other studies, SERIOUS TRAE OCCURRED EARLY IN THE COURSE OF TREATMENT AND RARELY AFTER THE FIRST TWO DOSES.

**Conclusion:** Serious treatment related adverse events are uncommon with grass-pollen SLIT and rarely occur after the first two doses. However, we describe a case of serious reaction treated with epinephrine after the fifth dose of GRASTEK.

**Consent to publish:** Consent to publish was obtained.

### A29 Hyperpigmented skin lesions, pruritus and anaphylaxis

#### Pascale Dupuis^1^, Lori A. Connors^1,2^

##### ^1^Department of Medicine, Dalhousie University, Halifax, NS, Canada, ^2^Allergy and Immunology, Dalhousie University, Halifax, NS, Canada

###### **Correspondence:** Pascale Dupuis

*Allergy, Asthma & Clinical Immunology* 2017, **13(Suppl 1)**:A29

**Background:** Mast cells are well known for their role in allergic reactions. When activated, they release multiple mediators of inflammation, a process termed degranulation. This process is thought to cause signs and symptoms such as flushing, urticaria, gastrointestinal symptoms and anaphylaxis. Mastocytosis is defined by abnormal mast cell proliferation and accumulation in various tissues. This is a case of mastocytosis presenting with recurrent anaphylaxis and pruritic skin lesions, which highlights the importance of early recognition and treatment of the disease.

**Case presentation:** A 58 year old male was referred to the allergy clinic after experiencing an episode consistent with idiopathic anaphylaxis. His past medical history was significant for a 5-year history of skin lesion; previously diagnosed as a dermatitis, prior anaphylaxis from shellfish and chronic gastrointestinal symptoms. His physical exam revealed hypergimented skin lesions with central telangiectasia typical of urticaria pigmentosa. Darier’s sign, urtication of the skin when lesions are stroked, was positive. Tryptase levels were elevated on three separate occasions. Skin biopsies showed numerous abnormal mast cells and telangiectasia macularis eruptiva perstans. A bone marrow biopsy revealed diffuse spindle-shaped mast cells. DNA testing expressed a KIT Asp816Val mutation, a proto-oncogene that entails mast cell hyperplasia. A diagnosis of indolent systemic mastocytosis was made. The patient currently takes anti-histamines and the pruritus has resolved. He has not experienced further episodes of anaphylaxis.

**Discussion:** Diagnosis and treatment of mastocytosis decreases health care utilization and costs. Visits to health care professionals for episodes of idiopathic anaphylaxis can be avoided when the disease is adequately controlled.

**Conclusion:** Clinicians should be aware of presenting symptoms of mastocytosis and refer to allergy and immunology when concern of mastocytosis exists.

**Consent to publish:** Consent to publish was obtained.

### A30 Severe and prolonged peri-operative anaphylaxis secondary to chlorhexidine-coated central venous catheter insertion

#### Michael N. Fein, Joseph Shuster

##### Department of Clinical Immunology and Allergy, McGill University, Montreal, QC, H3G 1A4, Canada

###### **Correspondence:** Michael N. Fein

*Allergy, Asthma & Clinical Immunology* 2017, **13(Suppl 1)**:A30

**Background:** Chlorhexidine is a widely used topical antiseptic which can be found in an increasing number of commercially available products. It is also impregnated in certain medical materials including central venous catheters, pleural tunneled catheters, and Foley catheters. Adverse reactions to chlorhexidine include delayed-hypersensitivity reactions as well as increasing reports of immediate IgE-mediated reactions [1, 2].

**Case presentation:** A 68 year-old male with past medical history of primary biliary cirrhosis, metastatic cholangiocarcinoma with previous Whipple surgery, and anastomotic biliary strictures, presented with severe peri-operative anaphylaxis during a planned revision hepatojejunostomy. After induction with midazolam, propofol, rocuronium, lidocaine, ampicillin, ceftriaxone, and fentanyl he developed generalized urticaria, mild airway edema without bronchospasm, and severe hypotension requiring epinephrine and vasopressors. His surgery was aborted. He had no previous history of drug allergies or food allergies and had undergone multiple previous surgeries without incident.

Following this episode, skin prick testing to penicillin and graded challenges to ceftriaxone and amoxicillin were performed and were negative. Local anesthetic testing was also negative.

Unfortunately, repeat surgery without antibiotics and using different induction agents resulted in recurrent severe peri-operative anaphylaxis with refractory hypotension immediately following insertion of a central venous catheter impregnated with chlorhexidine. Induction medications included propofol, fentanyl, succinylcholine, desflurane, glycopyrrolate, and lidocaine. Tryptase drawn in the operating room was 40.4 µg/L and later the same day was 6.8 µg/L.

Skin prick testing and intradermal testing was performed to all medications he received peri-operatively and to common induction agents including succinylcholine, cistracurium, rocuronium, fentanyl, midazolam, propofol, and lidocaine according to concentrations previously published [3]. All were negative except for skin prick testing to chlorhexidine gluconate 2% which was positive with a 7 mm wheal and 10 mm flare. The patient returned to the operating room three weeks later and had a successful surgery with the avoidance of all chlorhexidine products.

**Conclusion:** Peri-operative anaphylaxis is a challenging and potentially life-threatening clinical problem which demands a thorough evaluation of all possible culprit medications including topical antiseptics such as chlorhexidine.

**Consent to publish:** Consent to publish was obtained.

**References**

1. Silvestri et al. Chlorhexidine: uses and adverse reactions. Dermatitis. 2013;24:3.
2. Mirone et al. Identification of risk factors of severe hypersensitivity reactions in general anesthesia. Clin Mol Allergy. 2015;13:11.
3. Guyer et al. Comprehensive allergy evaluation is useful in the subsequent care of patients with drug hypersensitivity reactions during anesthesia. J Allergy Clin Immunol Pract. 2015;3:94–100.

### A31 Dramatic resolution of acute necrotizing eosinophilic myocarditis with a brief, high-dose steroid course

#### Hani Hadi, Brooke Polk, Nikita Raje

##### Division of Allergy and Immunology, Department of Pediatrics, Children’s Mercy Hospital, University of Missouri, Kansas City, MO, USA

###### **Correspondence:** Hani Hadi

*Allergy, Asthma & Clinical Immunology* 2017, **13(Suppl 1)**:A31

**Background:** Hypereosinophilic Syndrome (HES) represents a constellation of disorders characterized by increased production of eosinophils, both in tissues and peripherally. In this case report, we highlight the importance of early and aggressive treatment of acute eosinophilic necrotic myocarditis with corticosteroids in order to mitigate the potential for fatal consequences.

**Case description:** A previously completely healthy, athletic 14 year old female was admitted to the pediatric intensive care unit with a three day history of general malaise, chest pain and shortness of breath. Echocardiogram revealed severe hypertrophic cardiomyopathy with pericardial effusions and severely reduced ejection fraction. Despite treatment she continued to decompensate, such that by 72 h she had a near-fatal cardiac arrest, was intubated and put on extracorporeal membrane oxygenation (ECMO). She was placed at the top of the cardiac transplant list (1A). Cardiac biopsy performed during septostomy revealed severe extensive myocytic necrosis with eosinophilic infiltration.

A decision was made to trial her on methylprednisolone 25 mg/kg (1000 mg) for three days, followed by a slow oral taper. Within hours she exhibited improvement and by day three was extubated, off ECMO and ambulating. She was discharged home the subsequent week with completely normal cardiac function. Her serum eosinophil count, which had peaked at 12.10, was 0.18 at discharge (normal < 0.50 × 10^3^ cells/mcl).

**Discussion:** This case highlights the importance of early and aggressive treatment with corticosteroids in patients with cardiac involvement secondary to hypereosinophilia. Current literature has no established benchmark for corticosteroids dosing in acute myocardial necrosis secondary to hypereosinophilia, especially in the pediatric population. We have found that a dose of 25 mg/kg/day for three days with subsequent taper resulted in dramatic and rapid clinical improvement. This patient went from ECMO and the cardiac transplant list to being clinically asymptomatic and home within one week.

**Consent to publish:** Consent to publish was obtained.

### A32 A pediatric case of selective fixed drug eruption to amoxicillin

#### Roxane Labrosse^1^, Philippe Bégin^1,2^, Louis Paradis^1,2^, Anne Des Roches^1^, Jonathan Lacombe-Barrios^1^

##### ^1^Pediatric Allergy and Clinical Immunology, CHU Sainte-Justine, University of Montreal, QC, Canada, ^2^Allergy and Clinical Immunology, CHUM, University of Montreal, QC, Canada

###### **Correspondence:** Roxane Labrosse

*Allergy, Asthma & Clinical Immunology* 2017, **13(Suppl 1)**:A32

**Background:** Fixed drug eruption (FDE) is a distinctive type of delayed drug hypersensitivity reaction that presents with circumscribed skin lesions recurring at the same location upon exposure to the culprit drug. Appropriate testing to confirm diagnosis is still debated, and evaluation of reactivity to others agents with similar chemical structure is poorly documented. We present a rare case of FDE to amoxicillin reproducible with a graded drug provocation test (DPT) in which we also assed cross-reactivity with other beta-lactams.

**Methods:** Investigation was first performed by skin prick tests with penicilloyl-polylysine (PPL), benzylpenicillin (BP) and amoxicillin, and intradermal tests with PPL and BP. Subsequently, the patient underwent successive DPT to amoxicillin, penicillin V potassium and cefprozil in the allergy outpatient clinic with an interval of 4–6 weeks between each test. If negative, each DPT was followed by a four-day home DPT. Finally, patch testing with penicillin V potassium and amoxicillin was performed.

**Results:** We report a case of a 4-year-old boy who presented with a clinical picture compatible with FDE with well-demarcated oval lesions on the base of the neck, the manubrium, and the inner thigh the second day of amoxicillin treatment for an otitis media. Prick and intradermal skin testing were all negative with a positive histamine control. Amoxicillin DPT (45 mg/kg) showed a delayed positive response 4 h after exposure with a recurrence of the lesions at the same initial sites. The lesions resolved within two days with topical corticosteroid treatment. Subsequent DPT with penicillin V (25 mg/kg) and cefprozil (15 mg/kg) were negative. Finally, extra and intralesional patch testing were negative.

**Conclusions:** We describe the first pediatric case of a selective FDE to amoxicillin tolerating penicillin V potassium and cephalosporins with identical lateral chains. Moreover, our case strengthens the idea that DPT is the most reliable test for FDE diagnosis.

**Consent to publish:** The authors consent to publish this abstract in the AACI Journal. Written informed consent for publication was obtained from the patient.

### A33 An adverse reaction to omalizumab in a severe asthmatic patient

#### Sanju Mishra^1^, Gina Lacuesta^2^, Meredith Chiasson^3^, Babar Haroon^4^

##### ^1^Department of Medicine, Dalhousie University, Halifax, NS, Canada, ^2^Department of Medicine, Division of General Internal Medicine (Allergy and Immunology), Dalhousie University, Halifax, NS, Canada, ^3^Department of Medicine, Division of Respirology, Dalhousie University, Halifax, NS, Canada, ^4^Department of Critical Care and Department of Medicine, Division of General Internal Medicine, Dalhousie University, Halifax, NS, Canada

###### **Correspondence:** Sanju Mishra

*Allergy, Asthma & Clinical Immunology* 2017, **13(Suppl 1)**:A33

**Background:** Omalizumab is a humanized recombinant monoclonal antibody approved for treatment of moderate to severe allergic asthma. Adverse reactions include localized skin reactions most commonly; however, anaphylaxis is reported in the literature.

**Case presentation:** LM is a 45-year-old female with severe allergic asthma. She has required frequent hospitalizations and courses of steroids. LM has CT findings and spirometry concerning for asthma-COPD overlap syndrome (ACOS) with triggers of cigarette smoke, mould and cat dander at home. Prescribed respiratory medications include: tiotropium bromide, mometasone furoate/formoterol fumarate, montelukast, fluticasone furoate, and salbutamol. Ipratropium bromide is used during exacerbations. LM was prescribed omalizumab due to suboptimal ACOS control and a serum IgE level of 260. LM had recently finished a steroid taper and experienced dry cough, but returned to her baseline respiratory status before initiating omalizumab. Thirty minutes after receiving omalizumab LM experienced mild, transient pruritus in bilateral hands that resolved spontaneously. She then had acute dyspnea, wheezing and mild abdominal pain. There was no urticaria, angioedema, or hypotension, and her initial oxygen saturation was 99% on room air. She received IM epinephrine, salbutamol, cetirizine and prednisone and was transferred to the emergency department. In triage she was hypoexemic and treated for an asthma exacerbation under the care of internal medicine.

**Discussion:** Given the concerning history of anaphylaxis a tryptase level was ordered and treatment of an asthma exacerbation was continued. LM improved to her baseline the next morning and was subsequently discharged with no rebound anaphylaxis. The episode is challenging as history is concerning for anaphylaxis, but tryptase was low and several asthma triggers were present.

**Conclusion:** The case illustrates the challenge of assessing a potential adverse reaction to omalizumab in a severe asthmatic patient. The role of subsequent skin prick testing and a future challenge to omalizumab remains unclear.

**Consent to publish:** Consent to publish was obtained.

### A35 New onset colitis in an adult patient with chronic granulomatous disease treated with hematopoietic stem cell transplantation

#### Kara Robertson^1^, Thomas Issekutz^2,3^, Desmond Leddin^1,4^, Stephen Couban^1,5^, Lori Connors^1,6^

##### ^1^Department of Medicine, Dalhousie University, Halifax, NS, Canada, ^2^Department of Pediatrics, Dalhousie University, Halifax, NS, Canada, ^3^Division of Immunology, IWK Health Centre, Halifax, NS, Canada, ^4^Division of Gastroenterology, QEII Health Sciences Centre, Halifax, NS, Canada, ^5^Division of Hematology, QEII Health Sciences Centre, Halifax, NS, Canada, ^6^Halifax Allergy and Asthma Associates, Halifax, NS, Canada

###### **Correspondence:** Kara Robertson

*Allergy, Asthma & Clinical Immunology* 2017, **13(Suppl 1)**:A35

**Background:** Chronic granulomatous disease (CGD) is a rare primary immunodeficiency characterized by recurrent life-threating bacterial and fungal infections and granuloma formation. CGD is caused by defects in NADPH oxidase, which results in the inability of phagocytes (neutrophils, monocytes and macrophages) to destroy certain microbes. Hematopoietic stem cell transplantation (HSCT) is the only established curative therapy for CGD.

**Case presentation:** A 23 year old male with a history of X-linked CGD underwent a reduced-intensity matched unrelated donor peripheral blood stem cell transplant in July 2015. In February 2016, he was admitted to hospital for nutritional support secondary to odynophagia and anorexia. An upper endoscopy revealed ulcers in his esophagus and he was initially treated with IV acyclovir until biopsies came back negative for HSV/CMV. Pathology report showed a pattern of widespread inflammatory changes. He was then started on mesalamine. Nitroblue tetrazolium (NBT) testing showed no evidence of CGD and a peripheral blood chimerism study (VNTR) showed 100% donor alleles. It was then thought he might have graft-versus-host disease (GvHD) and he was treated with IV steroids. The patient subsequently had several episodes of lower GI bleeding. A colonoscopy demonstrated patchy severe active colitis with ulceration and one granuloma. CMV, acid-fast bacilli and Epstein Barr virus testing was negative. Flow cytometry revealed CD4 15%, CD8 16%, CD56 59% and CD19 1%. He was started on IVIG at immunomodulatory dosing, his MMF was discontinued, and prednisone and tacrolimus doses were adjusted. Repeat colonoscopy one month later showed very severe disease with no improvement.

**Discussion:** This patient with chronic granulomatous disease presented with a new-onset gastrointestinal inflammation and ulceration. This was initially suggestive of a diagnosis of CGD colitis, a common GI manifestation of x-linked CGD. After allelic testing, GvHD was then suspected due to the widespread inflammation, and plethora of phagocytes and lymphocytes within the gastrointestinal lining. However, since this patient has presented with esophageal and colonic involvement, is post-HSCT, and allelic testing demonstrates entirely donor alleles, the possibility of Crohn’s disease secondary to HSCT cannot be ruled out. This case would be one of the first documented reports of colitis secondary to HSCT in a patient treated to cure CGD, but the possibility of an infectious etiology cannot be excluded.

**Conclusion:** A broad differential diagnosis is required for colitis presenting after HSCT and in a patient with pre-existing CGD. This case delineates the need for interdisciplinary care and describes a severe case of colitis after HSCT.

**Consent to publish:** Consent to publish was obtained.

### A36 C1-esterase Inhibitor as an acute phase reactant: are we missing the diagnosis of Hereditary Angioedema?

#### Adrienne Roos^1^, Amin Kanani^1^, Edmond S. Chan^2^

##### ^1^Division of Allergy and Clinical Immunology, Department of Medicine, University of British Columbia, St Paul’s Hospital, Vancouver, BC, Canada, ^2^Division of Allergy and Immunology, Department of Pediatrics, Faculty of Medicine, University of British Columbia, Children’s Hospital, Vancouver, BC, Canada

###### **Correspondence:** Adrienne Roos

*Allergy, Asthma & Clinical Immunology* 2017, **13(Suppl 1)**:A36

**Background:** Hereditary Angioedema (HAE) is a rare, potentially life threatening disease of intermittent soft tissue and mucous membrane swelling caused by low levels of C1-esterase inhibitor (C1-INH), a complement protein that regulates bradykinin production. C1-INH is an acute phase reactant, but the implications of this on the interpretation of the C1-INH assay are not known.

**Case presentation:** A 3-year-old girl whose father has HAE was referred for assessment. History revealed no angioedema attacks. Blood work showed low C1-INH functional level at 0.26 U (normal 0.7–1.3 U) and low complement C4 at 0.07 (normal 0.15–0.47), consistent with the diagnosis. The investigations were repeated upon assessment by a pediatric allergist as two abnormal values are required to confirm the diagnosis. The second C1-INH functional level was in the normal range at 0.76 U. The patient did have an upper respiratory tract infection at the time the sample was drawn. She went on to have a mild attack of angioedema involving a finger and a third C1-INH functional level, when well, was again low at 0.36 U with undetectable C4. Functional C1-INH was measured using Siemens Chromogenic assay.

**Discussion:** This is the first reported case of increased C1-INH level during viral illness, suggesting its role as an acute phase reactant. This has impact on how clinicians test patients for HAE, as blood work drawn during a viral infection may produce falsely normal or elevated C1-INH levels. Given the autosomal dominant nature of this disease, many family members get tested and missed diagnosis may occur. Further research needs to assess a larger population of patients to determine C1-INH role as an acute phase reactant.

**Conclusion:** Low level of C1-INH is required for the diagnosis of Hereditary Angioedema. If it is an acute phase reactant, testing during a time of illness may result in falsely elevated levels and the diagnosis of HAE may be missed.

**Consent to publish:** Consent to publish this case report was obtained from the patient and her father.

### A37 Disseminated Mycobacterium avian complex (MAC) infection secondary to neutralizing anti-interferon gamma antibody in a Vietnamese man

#### Adrienne Roos, Robert Schellenberg

##### Division of Allergy and Clinical Immunology, Department of Medicine, University of British Columbia, St Paul’s Hospital, Vancouver, BC, Canada

###### **Correspondence:** Adrienne Roos

*Allergy, Asthma & Clinical Immunology* 2017, **13(Suppl 1)**:A37

**Background:** Adult-onset, acquired immunodeficiency due to anti-interferon gamma (IFN-ϒ) antibody was first described in 2004 and is seen only in the East Asian population. Autoantibody against IFN-ϒ lead to defective cell-mediated immunity, making individuals susceptible to disseminated non-tuberculous mycobacterial (NTM) infections and other opportunistic infections that are recalcitrant to standard antimicrobial treatment. Lung, lymph node, skin, soft tissue and bone are the tissues most affected with NTM. Rituximab, an anti-B cell therapy, has shown promise in eliminating this autoantibody.

**Case presentation:** A 60-year-old previously healthy Vietnamese man presented with weight loss, non-productive cough, shortness of breath, fatigue, bony pain, hypercalcemia and multiple subcutaneous lesions and draining sinuses. Mycobacterium avium grew in sputum and he was treated for pulmonary MAC, but his condition continued to deteriorate. Imaging showed lesions in lung, spine, ribs, skull, pelvis, both humeri and diffuse lymphadenopathy. Biopsy of rib, lymph node and lung lesion revealed non-necrotizing granulomas negative for malignancy or acid fast bacilli. CD4 count was 264 cells/mm^3^, IgG 26.3 g/L, IgA 4.73 g/L (polyclonal increases), bone marrow reactive, and HIV status negative. Serum contained neutralizing anti-interferon IFN-ϒ antibodies (NIH-S.Holland). The patient was initiated on rituximab as per lymphoma protocol along with continued antimicrobial therapy. Follow-up after 1 cycle/4 courses of Rituximab revealed weight gain, increased energy, absence of bone pain, and resolution of subcutaneous nodules and sinuses. 8 months later, he demonstrated worsening bony lesions and hypercalcemia that stabilized with a further 3 doses of rituximab given 4 weeks apart.

**Discussion:** This is the first Canadian case report of this disease. Given the large Asian population in this country it is an important entity for clinicians to be aware of. Like other published cases in Asia and the USA, our patient improved significantly with the use of adjunct Rituximab but repeated courses are likely required to maintain suppression of anti-IFNɣ antibodies. Without such antibody suppressive therapy, the results using anti-tuberculous therapies alone are very poor.

**Conclusion:** Cell-mediated immunodeficiency due to neutralizing interferon-gamma autoantibodies presents in East Asian adults, predisposing them to disseminated NTM infections. Rituximab, a B-cell depleting treatment, shows promise as an adjunct to antimicrobial therapy.

**Consent to publish:** Consent to publish this case report was obtained from the patient.

### A38 Late presentation of possible terminal compliment deficiency with recurrent Neisseria meningitides infection

#### Lana Rosenfield^1^, Anna Cvetkovic^2^, Kevin Woodward^3^, Jaclyn Quirt^1^

##### ^1^Division of Clinical Immunology and Allergy, McMaster University, Hamilton, ON, Canada, ^2^Department of Internal Medicine, McMaster University, Hamilton, ON, Canada, ^3^Division of Infectious Diseases, McMaster University, Hamilton, ON, Canada

###### **Correspondence:** Lana Rosenfield

*Allergy, Asthma & Clinical Immunology* 2017, **13(Suppl 1)**:A38

**Background:** The complement system has an important role in fighting Meningococcal disease. Recurrent meningococcal infection can be a significant feature of patients with terminal complement deficiency. We describe a patient with recurrent Neisseria meningitides bacteremia and a persistently low CH50 suggesting a deficiency in a terminal complement protein.

**Case presentation:** A 56 year old caucasian female with past history of severe Neisseria meningitides meningitis and bacteremia at age 42 years of age, presented with a 2 day history of feeling unwell with vomiting and loose stools. On presentation she was found to have a purpuric rash similar to that that occurred with her previous Neisserial meningitis. She was afebrile and looked well, with no meningismus, but was hypotensive. Although initially suspected to have leukocytoclastic vasculitis, blood cultures grew Neisseria meningitides.

Immunodeficiency work-up was initiated due to the recurrence of meningococcus infection, demonstrating CH50 < 10 U/mL three days post-presentation. Repeat CH50 after 19 days was unchanged. Individual terminal complement levels are pending. She had not been vaccinated after her initial meningococcal infection.

**Discussion:** This case allows a review of the approach to a patient with recurrent Neisseria meningitides infection, and the characteristics of the CH50 test. Terminal compliment deficiency would explain the presentation of our patient with multiple meningococcal infections. We are currently awaiting the results of confirmatory testing for the exact complement protein implicated by obtaining levels from a specialized lab in the United States. This case is unique in that patients with terminal complement deficiency typically present earlier in life than our patient.

**Conclusion:** This is a case of likely terminal complement deficiency presenting with recurrent Neisseria meningitides infection later in life than typically seen. Confirmation of this diagnosis is pending.

**Consent to publish:** Consent to publish has been obtained from patient presented.

### A39 An allergic reaction after a large quantity of pistachios

#### Wade T. A. Watson^1,2^, Edson Castilho^2^, Jennifer A. Sullivan^2^

##### ^1^Division of Allergy, Department of Pediatrics, Dalhousie University, Halifax, NS, Canada, ^2^IWK Health Centre, Halifax, NS, Canada

###### **Correspondence:** Wade T. A. Watson

*Allergy, Asthma & Clinical Immunology* 2017, **13(Suppl 1)**:A39

**Background:** Most allergic reactions to nuts occur with small quantities. We report a case where a large amount of nut ingestion was required in order to trigger an allergic reaction.

**Case history:** A 14 year old male ingested 160 g (approximately 100) pistachios over 30–60 min. 60 min later, he developed periorbital angioedema, sore throat and difficulty talking. He was nauseated. He was taken to the nearest hospital and he received an antihistamine and oral prednisone. He did not receive epinephrine. His angioedema resolved over several days.

An epicutaneous test was positive to fresh pistachio (8 mm wheal). The patient did not wish to carry an epinephrine auto-injector, as he did not believe he was allergic. A food challenge was offered and accepted. A graded oral challenge to pistachios was performed (1/4, ½, 1, 2, 4, 8, 16 and 32 nuts). Fifteen minutes after ingesting 32 pistachios (22.5 g) he developed perioral redness. His blood pressure was normal. 30 min later, there was a blotchy rash on his torso. He complained about feeling tired and a lump in his throat. He was given epinephrine, 0.3 mg intramuscularly, cetirizine and prednisone. His symptoms rapidly improved. He was observed for 2 more hours. No other symptoms developed.

**Discussion:** This case illustrates the importance of recognizing that threshold doses for allergic reactions can vary widely between patients. It also brings up other questions. Should he strictly avoid pistachio? Does he need to carry an epinephrine auto injector? Will the threshold for allergic reactions stay at this level or decrease with further exposures?

**Consent to publish:** Consent to publish was obtained.

## Food Allergy/Anaphylaxis

### A42 Reflections on the use of epinephrine for anaphylaxis

#### Rishma Chooniedass^1,2^, Beverley Temple^1^, Donna Martin^1^, Allan Becker^2^

##### ^1^College of Nursing, Faculty of Health Sciences, University of Manitoba, Winnipeg, MB, Canada, ^2^Department of Pediatrics and Child Health, University of Manitoba, Winnipeg, MB, Canada

###### **Correspondence:** Rishma Chooniedass

*Allergy, Asthma & Clinical Immunology* 2017, **13(Suppl 1)**:A42

**Background:** Food allergies and anaphylaxis have no cure and are on the rise. Epinephrine is the primary recommended treatment for anaphylaxis. Children with life threatening food allergies live with the constant threat of a fatal reaction, and caregivers must be prepared to treat with an epinephrine auto-injector (EAI). Morbidity and mortality are associated with a delay or lack of epinephrine use. Despite this, use of epinephrine for anaphylaxis is still underutilized.

**Methods:** A consent form and short questionnaire were distributed to caregivers of children under the age of 12 diagnosed with a food allergy, prescribed an EAI and who experienced an anaphylactic reaction within the last 2 years. In-depth, informal, semi-structured interviews were audio-recorded and transcribed verbatim. Nvivo software was used and interpretive phenomenological analysis was performed.

**Results:** Ten caregivers participated (5 females) 32–46 years (mean age 41 years). Children ranged from 3 to 10 years, all with multiple food allergies. Every child regularly carried an EAI, and had experienced between 2 and 7 anaphylactic reactions. Epinephrine treatment from caregivers ranged from 1 to 6 times. Six main themes emerged: life challenges, isolation, anxiety, hesitation, guilt, and influence of health care. Caregivers explained the multiple life challenges and feelings of isolation. During reactions, caregivers felt anxiety and hesitation that lead to subsequent guilt. They were often unsure if it was anaphylaxis, and waited for the situation to spontaneously improve. Interestingly, many mentioned that handling reactions correctly gave them confidence to treat subsequent reactions, which was often influenced by what they had learned from healthcare professionals.

**Conclusion:** Despite the anxiety of experiencing a child’s reaction, handling reactions correctly provided participants with confidence to treat subsequent reactions. Witnessing the rapid effects of epinephrine and receiving positive support from health care providers further facilitated their ability to quickly react to anaphylaxis.

### A43 Needs assessment for allergy education mobile app: review of patient, parent and physician views

#### Victoria E. Cook, Christopher Mills, Elodie Portales-Casamar, Edmond S. Chan

##### Division of Allergy and Immunology, Department of Pediatrics, University of British Columbia, BC Children’s Hospital, Vancouver, BC, Canada

###### **Correspondence:** Victoria E. Cook

*Allergy, Asthma & Clinical Immunology* 2017, **13(Suppl 1)**:A43

**Background:** Studies suggest that there are a growing number of patients who self-perceive food allergy [1–5]. There is considerable public misconception on food allergy prevalence, definition and management [6]. There is a need for accessible, evidence-based information on food allergies. This study will provide content for a mobile app to engage/educate the public.

**Methods:** A convenience sample of family physicians completed a questionnaire on demographics, allergy complaints/referrals, patient signs/symptoms, public perceptions, and attitudes on mobile application use for patients concerned about allergic disease. We have completed one focus group of adolescents ages 10–17; two additional adolescent and three parent groups are planned. Open-ended questions explored trusted health information sources, information to be included in a mobile app, and features that would facilitate use and trustworthiness. Discussions were recorded and transcribed. Comparative methods will be used to categorize emerging themes.

**Results:** 17 family physicians completed the questionnaire. 71% (n = 12) reported allergy as a common presentation. 71% (n = 12) responded patients ‘often’ present without signs/symptoms of true allergy. The most common presenting signs/symptoms included: eczema, urticaria, nasal congestion, wheeze, anaphylaxis, family history and parental concern. All felt that patients have a poor understanding of food allergy. 100% (n = 17) felt patients would benefit from a mobile app and would recommend this. 95% (n = 16) felt this would reduce allergy referrals. Adolescents from our initial focus group supported this. They recommended this content: (1) allergy definition/distinction from ‘intolerance’, (2) true allergy signs/symptoms, (3) forms of allergy testing, and (4) treatment of true allergy. They felt an app developed by Allergy physicians and institutions would be trusted. They suggested easy-to-navigate interfaces, bright colours and animated characters to increase engagement.

**Conclusions:** These results support development of a mobile app to educate/engage the growing number of patients concerned about allergy, and support family physician counselling. Content encompassing several domains was recommended. Physician and institution development would improve user trust.

**References**

1. Government of Canada HCHP and FBFD. Food Allergies and Intolerances. 2004 Jul 26. http://www.hc-sc.gc.ca/fn-an/securit/allerg/index-eng.php. Accesed 2015 Sep 10.
2. Soller L, Ben-Shoshan M, Harrington DW, Fragapane J, Joseph L, St-Pierre Y, et al. Overall prevalence of self-reported food allergy in Canada. J Allergy Clin Immunol. 2012;129(S):AB234.
3. Luccioli S, Ross M, Labiner-Wolfe J, Fein SB. Maternally reported food allergies and other food-related health problems in infants: characteristics and associated factors. Pediatrics. 2008;122 (Suppl 2):S105–12.
4. Zuberbier T, Edenharter G, Worm M, Ehlers I, Reimann S, Hantke T, et al. Prevalence of adverse reactions to food in Germany—a population study. Allergy Eur J Allergy Clin Immunol. 2004;59(3):338–45.
5. Osterballe M, Hansen TK, Mortz CG, Høst A, Bindslev-Jensen C. The prevalence of food hypersensitivity in an unselected population of children and adults. Pediatr Allergy Immunol. 2005;16(7):567–73.
6. Gupta RS, Kim JS, Barnathan JA, Amsden LB, Tummala LS, Holl JL. Food allergy knowledge, attitudes and beliefs: Focus groups of parents, physicians and the general public. BMC Pediatr. 2008;8(1):36.

### A44 Near-fatal anaphylaxis caused by chlorhexidine-coated central venous lines during renal transplantation: a case series

#### Lisa W. Fu^1^, Alexander Ho^2^, Jeffrey Zaltzman^3^, Lucy Chen^3^ and Peter Vadas^1^

##### ^1^Division of Allergy and Clinical Immunology, Department of Medicine, University of Toronto, Toronto, ON, Canada, ^2^Department of Anesthesia, University of Toronto, Toronto, ON, Canada, ^3^Division of Nephrology, Department of Medicine, University of Toronto, Toronto, ON, Canada

###### **Correspondence:** Lisa W. Fu

*Allergy, Asthma & Clinical Immunology* 2017, **13(Suppl 1)**:A44

**Background:** Chlorhexidine is an effective antimicrobial agent commonly used in the perioperative setting. Allergic reactions to chlorhexidine include contact dermatitis, fixed drug eruptions, asthma and life- threatening anaphylaxis. We describe a cohort of patients who experienced severe or near-fatal anaphylaxis during surgery for kidney transplantation. Investigation of agents administered peri- operatively identified chlorhexidine as the trigger.

**Methods:** Patients were referred for investigation of severe peri-operative anaphylaxis in a tertiary care university teaching hospital. All drugs administered prior to the anaphylactic event were tested by prick and/or intradermal methods. Where appropriate, serologic testing was done for specific IgE by ImmunoCAP.

**Results:** A total of 7 patients were identified who had severe anaphylaxis immediately after insertion of chlorhexidine coated central venous lines in preparation for renal transplantation. All 7 (100%) patients were male. The mean age at time of reaction was 59.9 years (range 30–69 years). Cardiac arrest was seen in 3 (43%) patients, hypotension in 3 (43%) patients, and wheezing and desaturation in 1 patient. Anaphylaxis severity scores were grade 3 (severe) in 100%. Post-anaphylaxis tryptase levels were measured in 3 patients and were all elevated (range 27.3 to >200 UG/L). All patients tested positive for chlorhexidine by one or more of skin prick test, intradermal test or serum chlorhexidine specific IgE. Other causes of intraoperative anaphylaxis were ruled out by testing.

**Conclusions:** The postulated route of sensitization is via repeated disinfection of fistula sites with chlorhexidine in preparation for hemodialysis sessions. This study identifies a unique population at risk for fatal anaphylaxis triggered by chlorhexidine coated central venous lines. High risk patients should be pre-screened and tested prior to elective surgery to identify those patients in whom strict peri-operative avoidance of chlorhexidine exposure is required.

### A45 Drug-induced anaphylaxis visits: a 4-year follow-up study in two Emergency Departments in Montreal

#### Sofianne Gabrielli^1^, Ann Clarke^2^, Harley Eisman^3^, Judy Morris^4^, Lawrence Joseph^5^, Sebastien LaVieille^6^, Moshe Ben-Shoshan^1^

##### ^1^Division of Pediatric Allergy and Clinical Immunology, Department of Pediatrics, McGill University Health Center, Montreal, QC, Canada, ^2^Division of Rheumatology, Department of Medicine, University of Calgary, Calgary, AB, Canada, ^3^Department of Emergency Medicine, Montreal Children’s Hospital, McGill University Health Centre, Montreal, QC, Canada, ^4^Department of Emergency Medicine, Hôpital du Sacré-Coeur, Montreal, QC, Canada, ^5^Department of Epidemiology and Biostatistics, McGill University, Montreal, QC, Canada, ^6^Food Directorate, Health Canada, Ottawa, ON, Canada

###### **Correspondence:** Sofianne Gabrielli

*Allergy, Asthma & Clinical Immunology* 2017, **13(Suppl 1)**:A45

**Background:** Currently there is sparse data on demographic and clinical characteristics of drug-induced anaphylaxis. We aimed to assess the percentage, demographics, clinical characteristics, and management of drug-induced anaphylaxis cases treated in a pediatric and an adult Emergency Department (ED).

**Methods:** Over a 4-year period, children and adults presenting to the Montreal Children’s Hospital and Hôpital du Sacré-Coeur ED with anaphylaxis were recruited as part of the Cross-Canada Anaphylaxis Registry (C-CARE). A standardized data entry form documenting symptoms and triggers of anaphylaxis was collected.

**Results:** From June 2012 to May 2016, 45 pediatric patients presented to the ED with drug-induced anaphylaxis of which 4.4% (95% CI 1, 10.7) of reactions were severe. More than half the children (57.8% [95% CI 42.8, 72.8%]) were treated with epinephrine. During this time, 65 adult patients presented to the ED with anaphylaxis to drugs of which 16.2% (95% CI 7.6, 26.3%) of the reactions were severe. Half of the patients (53.8% [95% CI 41.4, 66.3%]) were treated with epinephrine. 72.7% (95% CI 56.7, 88.8%) of children and 60.0% (95% CI 39.4, 80.6%) of adults had seen an allergist after the ED visit. Despite increasing percentage of anaphylaxis at the Montreal Children’s Hospital of 0.21% (95% CI 0.15, 0.27) over 4 years, the percentage of drug-induced anaphylaxis showed no significant change (−2.8% [95% CI −7.3, 1.7%]). While at Hôpital du Sacré-Coeur, the percentages of both anaphylaxis and drug-induced anaphylaxis showed no significant change from 2012 to 2016 (−0.0062% [95% CI −0.048, 0.036] and −1.2% [95% CI −14.7, 12.3%], respectively).

**Conclusions:** Given the constant percentage of drug-induced anaphylaxis visits and that almost 50% of reactions in children and adults were not treated with epinephrine, educational programs prompting the use of epinephrine in drug-induced anaphylaxis are required. Additionally, it is crucial to assess all drug-induced anaphylaxis by an allergist appropriately to avoid mislabeling of patients.

### A46 Daily peanut introduction in peanut-allergic patients with high reaction thresholds: a case series

#### François Graham^1,2^, Anne Des Roches^2^, Jonathan Lacombe-Barrios^2^, Louis Paradis^1,2^, Philippe Bégin^1,2^

##### ^1^Department of Allergy and Immunology, Centre Hospitalier de l’Université de Montréal Hôpital Notre-Dame, Montréal, QC, Canada, ^2^Department of Pediatrics, Allergy and Immunology Division, Centre Hospitalier Universitaire Sainte-Justine, Montréal, QC, Canada

###### **Correspondence:** François Graham

*Allergy, Asthma & Clinical Immunology* 2017, **13(Suppl 1)**:A46

**Background:** The past decade has seen a shift in paradigm towards oral introduction as a mean for tolerance. Many patients that react to oral food challenges (OFC) do so at a high threshold, and could benefit from daily introduction of foods without the need for formal oral immunotherapy (OIT). We hypothesized that introduction of peanuts in the diet of peanut-allergic patients with a high reactivity threshold would be safe and would promote tolerance.

**Methods:** Starting in 2015, patients with high reaction thresholds to peanuts on OFC at our center were invited to introduce low doses of peanuts (at least one peanut) at home on a daily basis and were followed every 3 months to assess tolerance and to increase daily dosages. A high reaction threshold was defined as a non-systemic reaction over 1 peanut (240 mg of peanut protein) or a systemic reaction over 5 peanuts (1200 mg of peanut protein). OFCs performed between 2012 and 2016 were examined to determine the rate of such high threshold reactors in our population. Previous high threshold reactors were invited to repeat OFCs to act as a reference cohort and to assess natural evolution.

**Results:** We introduced peanuts in the diet of 14 patients with high threshold reactions. The only adverse events reported were mild oral pruritus or abdominal pain, which could be prevented with oral antihistamines.

The rate of peanut OFC reactions since January 2012 was 14.2% (49/346). Of these patients, 71.4% (35/49) reacted to high amounts of peanuts.

**Conclusion:** In patients with high threshold reactivity, daily low amounts of peanuts can be introduced safely. This approach can be easily applied in general practice without needing the complex infrastructure of OIT protocols. Longer follow up of patients and of reference cohorts are needed to measure long-term outcomes.

### A48 Likelihood of sensitization to alternate ingredients in patients evaluated for chocolate allergy

#### Hani Hadi^1^, Charles Barnes^1^, Jay Portnoy^1^, Vincent Stagg^2^

##### ^1^Division of Allergy and Immunology, Department of Pediatrics, Children’s Mercy Hospital, University of Missouri, Kansas City, MO, USA, ^2^Health Services and Outcomes Research, University of Missouri, Kansas City, MO, USA

###### **Correspondence:** Hani Hadi

*Allergy, Asthma & Clinical Immunology* 2017, **13(Suppl 1)**:A48

**Background:** Chocolate is prepared from the cocoa seed *Theobroma cacao*. Public perception of chocolate allergy is high, despite the medical literature reporting IgE mediated reactions to cocoa seed as very rare. Given that chocolate is prepared from common allergenic ingredients, we sought to determine whether patients who are evaluated and test negative for chocolate in the form of IgE sensitization to cocoa seed are more likely to exhibit milk, peanut or tree nut sensitization.

**Methods:** A retrospective analysis of food specific IgE (sIgE) panels over a 12 year span at our institution was performed. SIgE values to cocoa seed, milk, peanut, almond, Brazil nut, cashew, chestnut, hazelnut, macadamia, pecan, pistachio and walnut were obtained using blood samples analyzed via the ImmunoCap method (Phadia, Uppsala, Sweden). A positive result indicating sensitization was defined as a sIgE of ≥0.35 kU/L.

**Results:** Results from 5849 food specific IgE panels were retrieved from clinical records. 447 patients had testing to cocoa seed performed, of which 433 samples tested negative (sIgE < 0.35 kU/L). Patients who tested negative to chocolate were significantly more likely to exhibit sensitization to tree nut, peanut or milk (p < 0.0001, Fisher’s Exact test). 40% exhibited sensitization to hazelnut, 37% to pistachio, 30% to pecan and 27% to peanut and milk. The likelihood of having any tree nut sensitization was 24%.

**Conclusion:** Patients who have sIgE testing to chocolate in the form of cocoa seed are significantly more likely to exhibit sensitization to tree nut, peanut and milk than chocolate itself. Among the allergens evaluated, hazelnut sensitization was more common than other tree nuts, peanut or milk. In patients who present with concerns surrounding possible chocolate allergy, we suggest evaluation of tree nut—particularly hazelnut—and to a lesser extent peanut and milk allergy rather than cocoa seed itself.

### A52 Egg sensitization is associated with delayed introduction of peanut in children who are not sensitized to peanut

#### Elinor Simons^1^, Diana Lefebvre^2^, David Dai^2^, Allan B. Becker^1^, Stuart E. Turvey^3^, Padmaja Subbarao^4^, Piushkumar Mandhane^5^, Malcolm Sears^2^

##### ^1^Section of Allergy and Immunology, Department of Pediatrics and Child Health, University of Manitoba, Winnipeg, MB, Canada, ^2^Division of Respirology, Department of Medicine, McMaster University, Hamilton, ON, Canada, ^3^Department of Pediatrics, British Columbia Children’s Hospital, Vancouver, BC, Canada, ^4^Department of Pediatrics, The Hospital for Sick Children, Toronto, ON, Canada, ^5^Division of Pediatric Respirology, Pulmonary and Asthma, Edmonton, AB, Canada

###### **Correspondence:** Elinor Simons

*Allergy, Asthma & Clinical Immunology* 2017, **13(Suppl 1)**:A52

**Background:** Delayed introduction of peanut has been shown to increase the risk of peanut allergy among children at high risk of peanut allergy, including children with egg allergy. We evaluated the association between sensitization (a positive skin prick test) to other highly-allergenic foods and delayed introduction of peanut in children without sensitization to peanut in an unselected Canadian cohort.

**Methods:** Caregivers of participants in the Canadian Healthy Infant Longitudinal Development (CHILD) Study prospectively reported their child’s age of dietary introduction to peanut in the first 3 years of life and frequency of peanut consumption at age 3 years. At ages 1 and 3 years, the children underwent skin prick testing for sensitization to peanut and other food allergens and assessment by a pediatric allergist or trained researcher for food allergies. We evaluated delayed peanut introduction among children who were sensitized to other highly allergenic foods, such as milk and egg, but not sensitized to peanut.

**Results:** Among children with negative skin prick tests to peanut at age 1 year, some children were sensitized to egg (5.52%) and milk (1.37%). Children who were not sensitized to peanut at age 1 year were more likely to have delayed the introduction of peanut until after age 18 months if they were sensitized to egg (OR 1.52, 95% CI 1.04–2.21). They were also still more likely to be avoiding peanut at age 3 years if they were sensitized to egg at ages 1 year (OR 2.45, 95% CI 1.66–3.62) or 3 years (OR 2.60, 95% CI 1.27–5.33), even if they had never had a reaction to peanut (OR 2.14, 95% CI 1.25–3.67).

**Conclusion:** Avoidance of peanut in egg-sensitized children without sensitization to peanut may reflect caregiver concern about introducing highly allergenic foods. Improved knowledge translation is needed to personalize food-allergen avoidance recommendations for children.

**Acknowledgements:** This study would not have been possible without the commitment and generosity of the CHILD participants and their families. We acknowledge the CHILD Study Investigators for their contributions. The CHILD study is supported by AllerGen NCE and the Canadian Institute of Health Research. This research is supported by the Children’s Hospital Research Institute of Manitoba and the University of Manitoba.

### A53 Prescription patterns of epinephrine auto-injectors for treatment of anaphylaxis in Manitoba children

#### Herman Tam, F. Estelle R. Simons, Elinor Simons

##### Department of Pediatrics and Child Health, University of Manitoba, Winnipeg, MB, Canada

###### **Correspondence:** Herman Tam

*Allergy, Asthma & Clinical Immunology* 2017, **13(Suppl 1)**:A53

**Background:** Epinephrine is the first-line medication for treatment of anaphylaxis. There are limited data pertaining to recent dispensing patterns of epinephrine auto-injectors (EAIs).

**Methods:** Using the Drug Programs Information Network (DPIN) provincial administrative pharmaceutical claims database, we analyzed dispensing data for EAIs among 0- to 16-year-olds from January 1, 2011 to March 31, 2015. We identified the number and percentage of children and teenagers for whom at least one EAI was dispensed. We evaluated the appropriateness of the EAI dose (0.15 mg or 0.3 mg) for the child’s age (25 kg ~97th percentile for age 5 years and 30 kg ~ rd percentile for age 12 years). We also evaluated whether concomitant prescription of one or more asthma medication affected the risk of dispensing a larger-than-expected or smaller-than-expected dose.

**Results:** EAIs were dispensed 25,562 times for 8998 children (2.5% of the pediatric population). EpiPen and Allerject were the two most common EAIs dispensed (98.2%); 53.6% were 0.15 mg and 46.4% were 0.3 mg. Three percent of prescriptions may have been inappropriately dosed based on age (1.44% of children over age 12 years dispensed a 0.15 mg EAI, 95% CI 1.13–1.83% and 1.62% of children under age 5 years dispensed a 0.3 mg EAI, 95% CI 1.36–1.94%). Among children who had an asthma medication dispensed, children under age 5 years were not more likely to have a higher-than-expected dose (relative risk [RR] 1.38, 95% CI 0.97–1.98) and teenagers over 12 years were not more likely to have a lower-than-expected dose (RR 0.81, 95% CI 0.50–1.30).

**Conclusions:** Most EAIs were dosed appropriately based on the age of the child. Children with an asthma medication were not more likely to have an unexpectedly large or small dose prescribed. Factors accounting for dosing preferences may be important for improving the proportion of children who have their EAI prescription filled.

## Immunology

### A54 Lipopolysaccharide inhibits Interleukin-13-induced Eotaxin-3 in human airway epithelial cells

#### Dhaifallah Alotaibi, Harissios Vliagoftis

##### Pulmonary Research Group, Faculty of Medicine, University of Alberta, Edmonton, AB, Canada

###### **Correspondence:** Dhaifallah Alotaibi

*Allergy, Asthma & Clinical Immunology* 2017, **13(Suppl 1)**:A54

**Background**: Interleukin-13 (IL-13) is a central effector cytokine in asthma and promotes eosinophilic inflammation, airway hyper-responsiveness, mucus secretion, epithelial damage and fibrotic changes in the airways. Many of the IL-13 effects in the lung are through its interactions with the airway epithelium. IL-13 stimulates bronchial epithelial cells to release eotaxin-3 (CCL26) through the activation of signal transducer and activator of transcription 6 (STAT6). CCL26 is a potent chemoattractant for eosinophils, a hallmark of asthma. The effects of bacterial infections on airway eosinophilia are incompletely understood. There is evidence that microbial products through Toll Like Receptors (TLRs) activation affect the release of eosinophil chemotactic factors. Here we studied the effects of TLR4 activation on IL-13-induced CCL26 induction in airway epithelial cells.

**Methods**: We used LPS (TLR-4 ligand) to mimic the effects of bacterial insult on the airway epithelium. The human bronchial epithelial cell line BEAS-2B was stimulated with LPS (10 μg/ml) alone or in combination with IL-13 (20 ng/ml) for 24 h. RNA was extracted and CCL26 mRNA measured using quantitative reverse transcriptase polymerase chain reaction (qRT-PCR). STAT6 phosphorylation was measured by western blot.

**Results**: BEAS-2B cell activation with IL-13 strongly induced CCL26 mRNA expression (16.48 ± 6.05 fold over unstimulated cells, n = 7, p < 0.05). Simultaneous treatment of the cells with LPS inhibited IL-13- induced CCL26 expression (n = 7, p < 0.02). IL-13 induced STAT6 phosphorylation in BEAS-2B cells, which peaked at 30 min. This phosphorylation was attenuated when cells were activated by IL-13 in the presence of LPS.

**Conclusion**: LPS, a TLR4 ligand, attenuates the effects of IL-13 on CCL26 expression in airways epithelial cells. This effect may be dependent on LPS preventing STAT6 activation by IL-13.

### A55 Examining the role of soluble TLR2 in regulating the development of food allergy

#### Bassel Dawod^1^, Matthew C. Tunis^2^, Jean Marshall^1,2^

##### ^1^Department of Pathology, Dalhousie University, Halifax, NS, Canada, ^2^Department of Microbiology and Immunology, Dalhousie University, Halifax, NS, Canada

###### **Correspondence:** Bassel Dawod

*Allergy, Asthma & Clinical Immunology* 2017, **13(Suppl 1)**:A55

**Background**: Food allergy includes immune sensitization towards one or more food proteins, such as beta- lactoglobulin (βLG) that is a major allergen in cow’s milk. Milk contains several regulatory molecules, such as TGF-β, Vitamin-A, and microbiota that can enhance oral tolerance. Soluble toll-like receptor 2 (sTLR2) that acts as a decoy receptor is found in breast milk, but its exact role in oral tolerance is not well studied. Having shown previously that TLR2 activation can lead to tolerance disruption toward ovalbumin but not milk, we hypothesise that it could be due to sTLR2 in milk. This work aims to understand the impact of sTLR2 and other regulatory molecules in milk on the development of oral tolerance towards potential allergens.

**Methods**: Murine models of oral tolerance to cow’s milk, βLG, and ovalbumin were established. Oral tolerance was assessed in wild-type and TLR2-deficient mice through analysis of antigen-specific antibody responses after a systemic antigen challenge. The bioactivity of milk and recombinant sTLR2 in blocking the effects of a TLR2 activator (PAM3 CSK4) were assessed in vivo and in vitro. The development of antigen-specific Tregs and tolerogenic dendritic cells were also assessed. sTLR2 levels in commercial milk products were analysed by ELISA and bioassay.

**Results**: Oral administration of skim-milk, βLG, and ovalbumin was sufficient to enhance the development of tolerance, independent of TLR2. Tolerized mice produced less allergen-specific IgE. Soluble TLR2 was detected in cow’s milk and in commercial baby formulas, which was sufficient to block the bioactivity of PAM3 CSK4 in vitro similar to recombinant sTLR2. Furthermore, cow’s milk feeding was also able to enhance the development of ovalbumin-specific Treg cells and tolerogenic-dendritic cells in the mesenteric lymph node of mice.

**Conclusion**: Our results confirm an important role for milk-regulatory molecules, including sTLR2, in the development of oral tolerance, which could inform both allergy prevention and treatment strategies.

**Acknowledgments**: Funded by AllerGen NCE and Canadian Institutes of Health Research (CIHR).

### A56 The interleukin-4 (IL-4) and interleukin-21 (IL-21) in vitro assay distinguishes patients with more non-infectious complications of Common Variable Immunodeficiency (CVID)

#### Marylin Desjardins^1,2^, Marianne Béland^2^, Duncan Lejtenyi^1^, Jean-Phillipe Drolet^3^, Martine Lemire^4^, Christos Tsoukas^4^, Moshe Ben-Shoshan^1^, Francisco J.D. Noya^1^, Reza Alizadehfar^1^, Christine T. McCusker^1,2^, Bruce D. Mazer^1,2^

##### ^1^Department of Pediatrics, McGill University, Montreal, QC, Canada, ^2^Meakins-Christie Laboratories, Research Institute of McGill University Health Centre, Montreal, QC, Canada, ^3^Department of Medicine, Université Laval, Quebec, QC, Canada, ^4^Department of Medicine, McGill University, Montreal, QC, Canada

###### **Correspondence:** Marylin Desjardins

*Allergy, Asthma & Clinical Immunology* 2017, **13(Suppl 1)**:A56

**Background:** CVID can manifest with primarily infectious manifestations, or by autoimmunity, lymphoproliferation and/or malignancy. We previously described an in vitro assay [CD27^+^memory B-cells development and IgG production in response to IL-4/IL-21 stimulation] that distinguished two groups of CVID subjects. The aim was to assess if this assay could be a tool to differentiate clinical phenotypes of CVID.

**Methods:** We recruited 40 CVID patients at the McGill University Health Centre and Centre Hospitalier Universitaire de Québec with age/sex-matched healthy controls. Clinical complications of CVID were collected retrospectively using C-PRIMES registry. Proportions of complications were compared using contingency table and Fisher exact tests between CVID responders (R) and non-responders (NR) to IL-4/IL-21 in vitro assay. These results were subsequently analysed in comparison to a reference assay based on the percentage of class-switched (CS) memory B-cells from the EuroClass trial.

**Results:** Analysis of the clinical characteristics of the CVID R and NR groups demonstrated a distinctive pattern that predominated in the NR group. Both groups exhibited similar rates of infectious manifestations and bronchiectasis, but CVID NR had significantly more complications such as autoimmune cytopenia, splenomegaly and granuloma compared to CVID R (difference = 39.6%, p = 0.04). Only CVID NR had symptoms of chronic diarrhea/enteropathy. Using the cut off of ≤2% CS memory B-cells from the EUROClass trial, 40.0% of CVID NR who experienced non-infectious complications in our cohort would not have been identified (odds ratio 2.7, 95% confidence interval of 0.7–9.7) compared to odds ratio of 5.3, 95% confidence interval of 1.3–22.5 with the IL-4/IL-21 assay.

**Conclusions:** The IL-4/IL-21 assay can identify CVID patients who experienced less versus more non-infectious complications. This assay could be an additional tool to baseline B-cell subpopulations phenotyping to establish CVID prognostic and clinical monitoring and should be validated prospectively for this purpose.

**Acknowledgements:** Supported by AllerGen NCE, the Richard and Edith Strauss Foundation and Immunodeficiency Canada. The research project was approved by institutional review ethics boards and informed consents were obtained for patients and controls.

### A57 The effects of phthalate and toll-like receptor ligand co-exposure on whole blood innate immunology

#### Danay Maestre-Batlle^1^, Evelyn Gunawan^1^, Christopher F Rider^1^, Anette K Bølling^2^, Olga M. Pena^1^, Christopher Carlsten^1^

##### ^1^Department of Respiratory Medicine, University of British Columbia, Vancouver, BC, Canada, ^2^Department of Air Pollution and Noise, Norwegian Institute of Public Health, Oslo, Norway

###### **Correspondence:** Danay Maestre-Batlle

*Allergy, Asthma & Clinical Immunology* 2017, **13(Suppl 1)**:A57

**Background:** Phthalates, a plasticizer used in many common commercial products, are ubiquitous environmental contaminants and epidemiological studies suggest that phthalate exposure is associated with development or worsening of airway diseases. Dibutylphthalate (DBP) is a type of phthalate found in high concentrations in indoor air and appears to have a high inflammatory potential. Increasing interest is focused on altered innate immune responses that may increase the risk of allergic asthma. The activation of receptors on innate immune cells trigger the production of cytokines that influence adaptive immune responses. We aimed to understand how phthalate alters the systemic innate immune response by analyzing the co-exposure of DBP and toll-like receptor ligands in whole peripheral blood in vitro. These findings will inform our upcoming double-blinded, randomized controlled, crossover study in which an in vivo model will be employed.

**Methods:** Peripheral blood was exposed to DBP for 20 h followed by LPS challenge for 4 h. The cellular fraction was analyzed by flow cytometry to study surface markers such as CD16, CD24, CD9 and CD14 on neutrophils, eosinophils, lymphocytes and monocytes, respectively. The supernatant was used to detect cytokine expression by ELISA. Statistical significance was assessed by repeated measures one-way ANOVA with Dunnett’s correction.

**Results:** The median fluorescence intensity (MFI) of CD14 decreased significantly when stimulation with DBP + LPS (72 ± 10) was compared to the unstimulated control (258 ± 6, p < 0.001) and DBP only (160 ± 12, p < 0.01). MFI of CD24 increased when the co-exposure condition (333 ± 229) was compared to DBP only (237 ± 168, p < 0.01). TNFa expression following co-exposure (723 ± 88 pg/ml) increased compared to stimulation with LPS only (640 ± 95 pg/ml, p = 0.03).

**Conclusions:** Stimulation of peripheral blood with DBP and LPS alters the innate immune cellular and humoral response in vitro; next stage of our project will assess other relevant ligands (R848, PMA/Ionomycin).

### A58 Efficacy, safety, tolerability, and pharmacokinetics of human immune globulin subcutaneous, 20% (SCIG 20%): final analysis of a phase 2/3 study in patients with primary immunodeficiency disease (PIDD) in North America

#### Daniel Suez^1^, Isaac Melamed^2^, Iftikhar Hussain^3^, Mark Stein^4^, Sudhir Gupta^5^, Kenneth Paris^6^, Sandor, Fritsch^7^, Christelle Bourgeois^8^, Heinz Leibl^8^, Barbara McCoy^9^, Martin Noel^10^, Leman Yel^9^

##### ^1^Allergy, Asthma and Immunology Clinic, PA, Irving, TX, USA, ^2^IMMUNOe Health Centers, Centennial, CO, USA, ^3^Vital Prospects Clinical Research Institute, PC, Tulsa, OK, USA, ^4^Allergy Associates of the Palm Beaches, North Palm Beach, FL, USA, ^5^University of California at Irvine, Irvine, CA, USA, ^6^LSU Health Sciences Center, Children’s Hospital of New Orleans, New Orleans, LA, USA, ^7^Independent Consultant, Vienna, Austria, ^8^Shire, Vienna, Austria, ^9^Shire, Cambridge, MA, USA, ^10^Shire, Bannockburn, IL, USA

###### **Correspondence:** Martin Noel

*Allergy, Asthma & Clinical Immunology* 2017, **13(Suppl 1)**:A58

**Background:** We report final results from a study of SCIG20% in patients aged 2 years or older with PIDD in North America.

**Methods:** Epoch 1 (13 weeks): IgG 10% was administered intravenously (IVIG) at prestudy doses every 3–4 weeks (Q3W/Q4W). Epochs 2–4: SCIG 20% administered weekly (Epoch 2 [~12–16 weeks], 145% of the weekly equivalent Epoch 1 dose; Epoch 3 [12 weeks], dose adjusted per AUC assessments in Epochs 1–2; Epoch 4 [40 weeks], dose adapted individually per Epoch 3 IgG trough levels). The primary endpoint was the validated acute serious bacterial infection (VASBI) rate.

**Results:** Seventy-four patients aged 3–83 years received SCIG 20% and 67 completed; no patient discontinued SCIG 20% due to a serious adverse event (AE) or adverse reaction (AR). During SCIG 20% treatment (n = 74), 1 VASBI (rate = 0.012/year; *P* < 0.0001) was reported; the all-infection rate/patient-year was 2.41. Local ARs occurred in 23 out of 74 patients (rate = 0.022/infusion); all were mild (92.5%) and moderate (7.5%). In 4327 SCIG 20% infusions, the median infusion rate was 60 mL/h/site resulting in a <1-h median infusion time. A 30–59-mL volume/site was used in 67.4% of infusions, and 7.4% infusions employed a 60 mL or greater-volume/site without tolerability issues. Overall, 84.9% of infusions were administered using 2 or fewer infusion sites; 99.8% were completed without slowing the rate or interrupting/stopping administration. Ratio of geometric means of AUC/week for IgG with individualized SCIG 20% treatment over IVIG 10% Q3W/Q4W was 109% (90% CI 104–113).

**Conclusion:** In patients treated with SCIG 20%, VASBI and infection rates were low, and infusions—most administered using 2 or fewer sites—were well-tolerated at relatively high infusion rates.

### A60 Long-term clinical outcome in adenosine deaminase-deficient patients following treatment: a longitudinal single-centre experience (1985–2015)

#### Ori Scott^1^, Brenda Reid^2^, Adelle Atkinson^2^, Vy Hong-Diep Kim^2^, Chaim M. Roifman^2^, Eyal Grunebaum^2^

##### ^1^Department of Paediatrics, The Hospital for Sick Children, Toronto, ON, Canada, ^2^Division of Paediatric Immunology and Allergy, The Hospital for Sick Children, Toronto, ON, Canada

###### **Correspondence:** Ori Scott

*Allergy, Asthma & Clinical Immunology* 2017, **13(Suppl 1)**:A60

**Background:** Adenosine deaminase (ADA) deficiency is widely recognized as causing severe combined immune deficiency (SCID), with therapeutic options including enzyme replacement (ERT), hematopoietic stem cell transplant (HSCT) and gene therapy. A unique feature of this entity compared to other forms of SCID is the ubiquitous nature of ADA expression, leading to multi-organ disruptions not addressed by current therapies.

**Objective:** To investigate the systemic long-term sequelae in children with ADA deficiency.

**Methods:** We reviewed the charts of 20 children treated for ADA deficiency in our centre between 1985 and 2015. Children were included if they had survived >5 years following treatment. We collected data regarding treatment modality, molecular markers of immune reconstitution, growth parameters, and any medical diagnosis necessitating treatment.

**Results:** Eight patients included in the study had survived a mean of 16.5 years after therapy, of whom 5 underwent HSCT, 2 received ERT, and 1 treated with GT. The majority displayed improved immune function, with increase in lymphocyte counts, and evidence of antibody production. Clinical sequelae included: (1) growth stunt (<3rd percentile) in 4/8 patients; (2) 7/8 patients had neurologic abnormalities, including developmental delay, epilepsy, and hearing loss; (3) lung disease was confirmed in 5/8 patients, including asthma, bronchiectasis, and bronchiolitis obliterans; (4) metabolic disturbances, such as osteopenia, hypothyroidism, and dyslipidemia were diagnosed in 3/8 patients; (6) neoplasms, including non-Hodgkin’s lymphoma and dermatofirosarcoma protuberans, were diagnosed in 3/8 patients; (7) allergic/atopic phenomena such as eczema and allergic rhinitis were observed in 4/8 patients; (8) significant reflux was diagnosed in 4/8 patients, of whom 2 required G-tube insertion.

**Conclusion:** While all treatment modalities allow for immune reconstitution and survival, significant long-term morbidity affects all patients. This study highlights the need for multi-disciplinary follow-up of ADA-deficient patients following therapy, and for improved management options of this systemic metabolic disorder.

### A61 T-cell receptor rearrangement, peripheral blood eosinophilia, and solid tumors

#### Eiman AlSelahi^1^, Fernando Aleman^1^, Amber Oberle^1^, Mike Trus^2^, Parameswaran Nair^1^

##### ^1^Division of Respirology, Department of Pathology and Molecular Medicine, McMaster University, Hamilton, ON, Canada

###### **Correspondence:** Eiman AlSelahi

*Allergy, Asthma & Clinical Immunology* 2017, **13(Suppl 1)**:A61

**Background:** The T-cell receptor (TCR) genes (alpha, beta, delta, and gamma) are comprised of numerous, discontinuous coding segments that somatically rearrange to produce heterodimeric cell surface T-cell receptors, either alpha/beta (90–95% of T cells) or gamma/delta (5–10% of T cells). Distributive differences in both TCR sequence and genomic rearrangement fragment sizes can be detected by molecular techniques and is useful to determine if a population of T cells in conditions such as hyper-eosinophilic syndromes shows monoclonal or polyclonal features.

**Objectives:** To observe the association between TCR gene rearrangements and peripheral blood eosinophilia.

**Methods:** As part of a comprehensive immune-cytogenetic and hematology evaluation of patients with respiratory symptoms and leucocyte disorders (n = 48) in a newly established joint respirology-haemoncology clinic at the Firestone Institute for Respiratory Health, TCR gene rearrangements in peripheral blood was examined by PCR in 23 patients.

**Results:** 7 of the 23 patients (30%) were detected to have TCR-γ gene rearrangement. There was no difference in the proportion of patients with peripheral blood eosinophilia (>1500/µL) in those with the rearrangement (4/7) and those in whom the rearrangement was not detected (12/16). However, we observed that 4 of the 7 patients with the TCR rearrangements (3 with eosinophilia) had solid tumors of kidney, lung, colon or breast (with three of them developing the tumors after the rearrangement was detected). In one patient, the TCR gene rearrangement disappeared after the tumor was removed.

**Conclusion:** We suggest a careful evaluation of TCR gene arrangements associated with peripheral blood eosinophilia as a potential marker of solid organ tumors.

**Acknowlegements:** Dr. Nair was funded by the Canada Research Chair Program. Dr. AlSelahi was funded by the Ministry of Education, State of Kuwait.

## Other Allergy/Immunology

### A62 Patient demographics and real-world use of omalizumab for the treatment of chronic spontaneous/idiopathic urticaria in Canada: analysis of patient support program data

#### Gordon Sussman^1^, Amin S. Kanani^2^, Gina Lacuesta^3^, Olivier Chambenoi^4^, Sima Chiva-Razavi^4^

##### ^1^Division of Allergy and Clinical Immunology, St Michael’s Hospital, University of Toronto, Toronto, ON, Canada, ^2^Division of Allergy and Immunology, Department of Medicine, University of British Columbia, Vancouver, BC, Canada, ^3^Department of Medicine, Dalhousie University, Halifax, NS, Canada, ^4^Novartis Pharmaceuticals Canada Inc, Dorval, QC, Canada

###### **Correspondence:** Sima Chiva-Razavi

*Allergy, Asthma & Clinical Immunology* 2017, **13(Suppl 1)**:A62

**Background:** As there are limited publications on real-life use omalizumab in chronic idiopathic urticaria (CIU) in Canada, there is a need to generate data to inform us of patient characteristics and use of omalizumab. The study aims to describe patient demographics and treatment patterns of patients prescribed omalizumab in a real-world Canadian setting.

**Methods:** Following regulatory approval, a nationwide patient support program (PSP) was made available to all Canadian patients prescribed omalizumab. Baseline demographics and treatment information were collected for patients who provided consent, enrolled between August 26 2014 and April 21 2016; additional patient reported information was collected as of November 2015.

**Results:** The cohort included 1391 patients enrolled in the PSP who received at least one dose of omalizumab (71% women; average age: 46). Most patients (73%) were prescribed OMA 300 mg q4wks while 15% were prescribed 150 mg q4wks; 12% of patients were treated with other dosing regimens. Treatment history was reported by 191 patients; 70% reported being treated with ≥1 H1-antihistamine while 35% received montelukast. Prednisone was used in 24% of patients; use of cyclosporine was uncommon. Data on angioedema was available for 130 patients; 65% reported having a history of angioedema, 26% had not experienced angioedema, while 8% did not know. ER visits in the last year were reported by 137 patients; 55% reported not having visited the ER while the rest had been at least once. Average omalizumab treatment persistency is 13 months.

**Conclusion:** In a real-life world Canadian setting, omalizumab is prescribed to a population comparable to that of the Phase 3 clinical program. Treatment history and healthcare resource use were likely underestimated as data were patient reported and collected as of November 2015. There remains a need to further collect and disseminate real-world data to understand the effectiveness of omalizumab in a real-world setting.

### A64 The role of anesthesiologists in the management of patients with a suspected penicillin allergy

#### Savannah Grodecki^1^, Nikhil Joshi^2^, Peter Menikefs^3^, David Holt^3^, Teresa Pun^4^

##### ^1^Royal College of Surgeons, Dublin, Ireland, ^2^Department of Medicine, University of Manitoba, Winnipeg, MB, Canada, ^3^Department of Anesthesia, St Joseph’s Health Centre, Toronto, ON, Canada, ^4^Community Allergist, Toronto, ON, Canada

###### **Correspondence:** Savannah Grodecki

*Allergy, Asthma & Clinical Immunology* 2017, **13(Suppl 1)**:A64

**Background:** A self-reported history of penicillin allergy often results in the use of less effective and more expensive antibiotics. This practice also subjects patients to the complications of broader spectrum antibiotics and longer hospital stays. Phase 1 of our study aims to identify the barriers to referral amongst anesthesiologists.

**Methods:** A qualitative survey was completed by anesthesiology staff and senior residents. We questioned the perceived barriers to referral, if they thought a referral would be of benefit, and how a history of penicillin allergy alters their preoperative management.

**Results:** 15 responses were collected. 66.7% of respondents have never referred patients for a drug allergy evaluation, however 80% of anesthesiologists felt a referral would be beneficial. Barriers to referral included the potential for delay to surgery (33.3%) and the lack of an affiliated allergist to expedite a consult (33.3%). 86.6% of anesthesiologists change their preoperative antibiotic to vancomycin or clindamycin, while 13.3% rarely substitute an alternative for cefazolin.

**Conclusion:** While a large percentage of anesthesiologists believe a referral to an allergist for drug allergy testing is beneficial, only 33.3% have completed a referral in the past due to a host of systemic barriers. Phase 2 will work with anesthesiologists, surgeons and infectious disease at St. Joseph’s Health Centre to identify and refer all pre-operative patients with a penicillin allergy. Primary objectives include decreased costs of pre-operative antibiotics. Secondary objectives include the incidence of iatrogenic infections and length of stay.

### A65 Cord blood dendritic cell profiles and year one atopic manifestations in the Canadian Healthy Infant Longitudinal

#### Damian Tworek^1^, Raphael Hanna^1^, Delia Heroux^1^, Elli Rosenberg^1^, Leah Stiemsma^2^, Stuart Turvey^3^, Judah Denburg^1^

##### ^1^Division of Allergy and Clinical Immunology, Department of Medicine, McMaster University, Hamilton, ON, Canada, ^2^Department of Microbiology and Immunology, University of British Columbia, Vancouver, BC, Canada, ^3^Department of Pediatrics, University of British Columbia, Vancouver, BC, Canada

###### **Correspondence:** Raphael Hanna

*Allergy, Asthma & Clinical Immunology* 2017, **13(Suppl 1)**:A65

**Background:** Dendritic cells (DC) are important in innate and adaptive immune responses. Available data on cord blood (CB) DC phenotype with respect to allergic disease development are scarce. In this pilot study, we compared conventional and plasmacytoid CB DC cell surface receptor expression in CHILD cohort subjects with and without signs of atopy at 1 year of age. We hypothesized differential receptor expression profiles between atopic and non-atopic infants.

**Methods:** CB mononuclear cells from a randomly chosen subset of subjects participating in the CHILD Study were used to phenotype conventional (cDC) and plasmacytoid (pDC) CB DC compartments. Expression of toll-like receptors (TLR), epithelial derived cytokine receptors, co-stimulatory molecules and high affinity IgE receptors were measured by means of flow cytometry. Expression of CB DC receptors was compared between subjects with and without signs of atopy [positive skin prick test or physician diagnosed atopic dermatitis (AD)] at 1 year of age.

**Results:** TLR-9 expression on pDC was significantly lower (p < 0.05) in subjects with AD compared to infants without signs of atopy. In contrast, TLR-5 on cDC was higher in subjects with AD. FcεRI expression on pDC was significantly higher in subjects with a positive skin prick test (p < 0.01) or AD (p < 0.001) compared to subjects without atopy. There was a significantly higher expression of IL-33 receptor (ST-2) on pDCs from subjects with AD (p < 0.01) with a similar trend seen for cDC.

**Conclusion:** Lower pDC TLR-9 expression may indicate a weakened Th1 immune response, whereas increased expression of TLR-5, FcεRI, and ST-2 receptors suggest early biases favouring Th2 responses. These preliminary results indicate that specific CB DC phenotypes are associated with atopic development in the first year of life, and point to potential changes in innate and adaptive immunity which may pre-exist in utero in infants at risk of an allergic disease trajectory.

### A66 Cow’s milk elimination alone results in symptom and histologic improvement in a majority of children with Eosinophilic Esophagitis

#### Christopher Mill^1^, Timothy Teoh^3^, Preeti Zimmer^2^, Vishal Avinashi^2,3^, Edmond S. Chan^1,3^

##### ^1^Division of Allergy and Immunology, Department of Pediatrics, University of British Columbia, Vancouver, BC, Canada, ^2^Division of Gastroenterology, Hepatology and Nutrition, Department of Pediatrics, University of British Columbia, Vancouver, BC, Canada, ^3^Faculty of Medicine, University of British Columbia, Vancouver, BC, Canada

###### **Correspondence:** Christopher Mill

*Allergy, Asthma & Clinical Immunology* 2017, **13(Suppl 1)**:A66

**Background:** One treatment modality for Eosinophilic Esophagitis (EoE) involves the removal of offending dietary antigens. The traditional six-food-elimination diet has long been considered a standard treatment. However, this approach has shown suboptimal effectiveness (72%) despite it being very restrictive, and can negatively affect patients’ quality of life [1]. In 2012, Kagalwalla described an initial series of 17 patients who underwent cow’s milk elimination (CME) with 65% achieving clinical and histological remission (<15 mean peak eosinophils per high power field (eos/hpf)) [2]. This outcome has been further supported by a 2015 study showing 64% (n = 20) of patients achieving remission on a CME diet [3].

**Objective:** To describe the clinical and histological response of study subjects who have undergone a CME diet as a primary intervention.

**Methods:** This study was approved by The University of British Columbia/Children’s and Women’s Health Centre of British Columbia Research Ethics Board. A retrospective chart review was performed on EoE Registry patients that were followed in a multidisciplinary EoE Clinic at BC Children’s Hospital (BCCH), or seen in GI clinic. Data on follow up endoscopy and biopsy counts, symptoms as assessed at follow up visits and skin prick test results were collected to measure the effectiveness of a CME diet.

**Results:** Among 125 reviewed charts, 31 (25%) patients followed a CME diet. The majority (90%) of patients had high symptomatic improvement. Histologically, nearly three quarters (74%) of patients on a CME diet had decreased eosinophil counts after the intervention. For both responders and non-responders, the mean average peak eosinophil count decreased by 26 (Pre-treatment mean counts: 47.3 eos/hpf vs Post-treatment mean counts: 21.7 eos/hpf). Histologic remission (<15 eos/hpf) was achieved in 58% (18/31), with 16% (5/31) obtaining complete remission (0 eos/hpf). Patients who followed CME to a strict degree (i.e. reading labels and looking for hidden sources of milk protein) had a (63%, 14/22) chance of remission, compared to (44%, 4/9) who avoided only obvious sources of dairy (milk, cheese, yogurt, and creamy items). No differences in remission rates were seen with strict diets when compared with less strict diets (p = 0.39). There was no differential response to CME for those with atopy versus those without (p = 0.48). Skin prick testing to milk was not predictive of response to CME amongst the 14 patients who underwent testing.

**References**

1. Wechsler JB, Schwartz S, Amsden K, Kagalwalla AF. Elimination diets in the management of esosinophilic esophagitis. J Asthma Allergy. 2014;7:85–94.
2. Kagalwalla AF, Amsden K, Shah A, Ritz S, Manuel-Rubio M, Dunne K, et al. Cow’s milk elimination: a novel dietary approach to treat eosinophilic esophagitis. J Pediatr Gastroenterol Nutr. 2012;55(6):711–6.
3. Kruszewski PG, Russo JM, Franciosi JP, Varni JW, Platts-Mills TA, Erwin EA. Prospective, comparative effectiveness trial of cow’s milk elimination and swallowed fluticasone for pediatric eosinophilic esophagitis. Dis Esophagus. 2015;26.

### A67 Nasal inflammatory biomarkers in pediatric cystic fibrosis with and without nasal polyposis: a cross-sectional comparative study

#### Mihaela Paina^1^, Ahmed A. Darwish Hassan^2^, John Paul Oliveria^2^, Chris Olesovsky^2^, Gail Gauvreau^2^, Linda Pedder^1^, Paul K. Keith^2^

##### ^1^Department of Pediatrics, McMaster University, Hamilton, ON, Canada, ^2^Department of Medicine, McMaster University, Hamilton, ON, Canada

###### **Correspondence:** Mihaela Paina

*Allergy, Asthma & Clinical Immunology* 2017, **13(Suppl 1)**:A67

**Background:** Nasal polyposis (NP) complicates the course of cystic fibrosis (CF). Neutrophil esterase (NE) has been found to be high in the sputum of CF-patients. Eosinophil peroxidase (EPO) is prevalent in the sputum of non-CF-patients with NP. These inflammatory biomarkers have not previously been examined in the nasal secretions of CF-patients.

**Methods:** We studied 26 pediatric CF-patients. 16 patients had nasal polyposis (62%), as diagnosed by anterior rhinoscopy. Nasal lavage was obtained by modified Naclerio technique. Nasal inflammatory mediators (NE, EPO) were measured by ELISA. NE and EPO levels above median values were considered high. Data were analyzed by Chi square and t tests. P value <0.05 was considered to be statistically significant.

**Results:** High nasal neutrophil and NE levels tended to be more prevalent in CF-NP patients (62%) versus CF-non-NP patients (22%, p = 0.13). Nasal eosinophils and high EPO levels were less prevalent in both groups. CF-NP patients with high levels of NE were more likely to have a higher BMI (21 vs. 18 kg/m^2^, p = 0.04). CF-NP patients with high levels of EPO were more likely to have positive Aspergillus serum precipitins (24 vs. 0%, p = 0.04) and normal FEV1 (92 vs. 43%, p = 0.01). The proportion of positive allergy skin prick tests (50 vs. 70%, p = 0.29) and *Staphylococcus aureus* in sputum/throat cultures (54 vs. 56%, p = 0.93) was similar in CF-NP and CF-non-NP patients, respectively, irrespective of the NE and EPO levels.

**Conclusions:** CF-NP patients may constitute a clinical subgroup within the spectrum of the disease, with higher neutrophil and NE levels in nasal secretions and a significantly better nutritional status (as reflected by a higher BMI) compared to CF-non-NP patients. The subgroup of CF-NP patients with high EPO levels demonstrates higher exposure to Aspergillus and better pulmonary function. In conclusion, nasal NE and EPO could be useful in monitoring sinonasal disease in CF-patients.

**Acknowledgements:** This research study received funding from the Ontario Thoracic Society (Block Term Grant).

### A68 Stability of allergen extracts diluted in normal saline phenol (NSP), human serum albumin (HSA) or glycerin

#### Greg Plunkett, Michelle Bolner

##### ALK, Inc., Round Rock, TX, USA

###### **Correspondence:** Greg Plunkett

*Allergy, Asthma & Clinical Immunology* 2017, **13(Suppl 1)**:A68

**Background:** Our laboratory previously determined that the potency of diluted allergen extracts is dependent on concentration of protein and the type of diluents used in treatment vials. Treatment set vials (mixed allergens) and single allergen vials were serially diluted with NSP, HSA, 10 or 50% glycerin. Potency was defined as major allergen content and measured at baseline to 4 months. These results demonstrated that vials with protein content below about 30 μg/mL, regardless of allergen type, rapidly lost potency. Furthermore, potency was maintained by the addition of HSA. Since many maintenance vials are given longer expiration dating, this study increased testing for vials stored up to 24 months at 2–8 °C. This data is a continuation of our published experiments, demonstrating major allergen content can be maintained in vials containing single and mixed extracts.

**Methods:** Timothy Grass, Birch, Mixed Mite and Cat Hair extracts were used for this study. Equal parts of glycerinated allergen extracts were added to a 5 mL vial and dilutions made with NSP or added HSA (300 μg/mL). Each single allergen was separately serially diluted with NSP, HSA, 10 or 50% glycerin. All vials were stored at 2–8 °C during this study. Vial samples were tested for major allergen content by monoclonal ELISAs (ALK, Inc) after 24 months and compared with values at the time of mixing.

**Results:** More concentrated vials maintained allergen content in all diluents at 24 months. Diluted extract vials exhibited reduced protein content in NSP diluent, but protein content was maintained in the NSP + HSA vials.

**Conclusion:** This study shows that for four common allergens, mixed treatment maintenance vials retain major allergen content long enough to justify a use dating of 12 months. Additionally, dramatic loss of potency in lower protein containing build up vials is not observed in the presence of HSA.

### A69 Study of the effects of complementary and alternative medicine (CAM) on the practice of allergy in primary care and subspecialty settings: preliminary data from the subspecialty survey

#### Persia Pourshahnazari, Donald Stark

##### Department of Medicine, Division of Allergy and Clinical Immunology, University of British Columbia, Vancouver, BC, Canada

###### **Correspondence:** Persia Pourshahnazari

*Allergy, Asthma & Clinical Immunology* 2017, **13(Suppl 1)**:A69

**Background:** Interest in complementary and alternative medicine (CAM) has gained popularity [1]. An Ipsos-Reid survey of Canadians demonstrated that 73% of Canadians regularly take natural health products [2]. Allergy and immunology is especially targeted by CAM therapies, which frequently cite an ability to alter patients’ immune systems [3]. We seek to investigate the effect of CAM on the Canadian practice of allergy in both primary care and allergy/immunology subspecialty settings. We present here preliminary data from a subspecialty survey.

**Methods:** Email invitations to complete an anonymous online web survey were sent to Canadian allergists/immunologists. The survey consisted of 33 questions designed to collect data on practice demographics, patient/practitioner use of CAM, and the impact of CAM usage on daily clinical practice. Survey responses were analyzed for frequency of options selected.

**Results:** 48 responses were received, mainly from Ontario (48%). Most respondents practice full-time (83%) in a private/community setting (50%). 85% of those surveyed do not incorporate CAM into their own practice. Respondents felt that 11–40% of their patients had sought out CAM (69%), choosing predominantly Naturopathy (56%). Average patient expenditure on CAM was $100–$999 (58%), with most treatments lasting between 1 and 9 months (73%). Patients were thought to seek out CAM mainly due to a reluctance to take prescription medications (88%). Less than half of patients found CAM beneficial (79%). Patients most frequently voiced concerns regarding efficacy of treatments (65%). Typical patient visits amongst those using CAM were longer by up to 60 min (85%). There was no difference noted between investigations ordered, referrals to other specialties, or follow-up visits.

**Conclusions:** Preliminary survey data suggests that the use of CAM by patients has an impact on the practice of allergy/immunology by subspecialists in Canada. Final survey data will be analyzed further once collection is completed, then compared to data from the primary care setting.

**References**

1. Engler RJM, With CM, Gregory PJ, Jellin JM. Complementary and alternative medicine for the allergist-immunologist: Where do I start? J Allergy Clin Immunol 2009;123:309–16.
2. Natural Health Product Tracking Survey—2010 Final Report. http://epe.lacbac.gc.ca/100/200/301/pwgsctpsgc/por-ef/health/2011/135-09/report.pdf.
3. Engler RJM, Silvers WS, Bielory L. Complementary and alternative medicine education: Need for expanded educational resources for American Academy of Allergy, Asthma and Immunology members. J Allergy Clin Immunol. 2009;123(2):511–12.

### A70 Observational study of pollen counts in British Columbia from 2000 to 2015

#### Kateryna Vostretsova, Andrew Moses, Amin Kanani, Donald Stark

##### Faculty of Medicine, University of British Columbia, Vancouver, BC, Canada

###### **Correspondence:** Kateryna Vostretsova

*Allergy, Asthma & Clinical Immunology* 2017, **13(Suppl 1)**:A70

**Background:** Allergic diseases in Canada are on the rise and are associated with medical, societal and economic burdens. Pollens are the main trigger and cause of respiratory allergic conditions. Total pollen counts have been rising and have become more serious and of longer duration as a result of warmer temperatures and climate change. Knowledge of pollen counts and trends would allow for targeted patient allergy management.

**Methods:** We sought to identify major trends in pollen counts over the past 15 years in two major urban centers in British Columbia. Data was obtained from Aerobiology, a company responsible for outdoor pollen and fungal spore identification. Prevalent allergens known to cause the majority of allergic conditions including alder, grasses and Cladosporium were analyzed from 2000 to 2015. A literature review was undertaken to explore the role of these allergens and their effect on systemic allergic disease in the context of climate change.

**Results:** Alder is a major allergen in Vancouver and pollen counts have been increasing over the past 15 years. In Victoria, grasses predominate with data suggesting a shift towards an earlier grass pollen season and higher overall pollen counts while Alder pollen counts have been relatively stable. For both locations, the common mold, Cladosporium, is peaking later during its usual season further supporting the evidence of rising temperatures and longer warmer seasons.

**Conclusions:** Although Vancouver and Victoria are in close proximity, they have very different patterns of pollen distribution. These findings will help guide patient management and fuel further research in this area.

### A71 Retrospective review of beta lactam allergy prevalence in a referral population

#### Andrew Wakeman^1^, Alexander Singer^2^, Thomas Gerstner^3^, Elissa Abrams^3^

##### ^1^University College Dublin, Dublin, Ireland, ^2^Department of Family Medicine, University of Manitoba, Winnipeg, MB, Canada, ^3^Department of Pediatrics and Child Health, University of Manitoba, Winnipeg, MB, Canada

###### **Correspondence:** Andrew Wakeman

*Allergy, Asthma & Clinical Immunology* 2017, **13(Suppl 1)**:A71

**Background:** Penicillin allergies are over diagnosed with estimates of 10–20% prevalence. Research suggests that 90% of patients labeled beta lactam allergic tolerate penicillin upon assessment. In addition, most patients with true penicillin allergy will lose their sensitivity over time. The label ‘penicillin allergic’ is associated with poorer health outcomes and higher healthcare costs.

**Methods:** A retrospective chart review was performed for all evaluations of beta lactam allergy from January 2010 to June 2015 at a community allergy clinic. Testing performed was based on specialist recommendation following review of patient history and included intradermal testing and/or on-site oral drug provocation as deemed appropriate. Final outcomes were decided by a consensus of consultant investigators based on reaction history and results of skin and/or oral drug provocation testing.

**Results:** Our sample includes 306 referred patients with a history of a reaction to a beta lactam antibiotic. Mean age was 11.6 (SD 17.3); 49.5% of patients were male. Most reactions were to amoxicillin, and most reactions were reported as mild non-urticarial skin reactions. There were only 1/106 (0.01%) positive intradermal tests. Only 2/191 (0.01%) patients reacted with an oral drug provocation test with symptoms consistent with IgE-mediated hypersensitivity. Six patients were not tested due to original reaction symptoms suggestive of a serious delayed reaction. Four patients had delayed non- urticarial exanthems after oral drug provocation testing. Overall, 294 patients (96.1%) were determined not to require future avoidance of any beta lactam antibiotic and increased risk of anaphylaxis was ruled out in 304 (99.3%) cases.

**Conclusions:** Patients with documented beta lactam allergy were rarely allergic upon evaluation. This supports arguments for strategic widespread testing and delabelling of ‘penicillin allergic’ outpatient populations, particularly for pediatric patients. Elective allergy assessment is an excellent opportunity to simultaneously improve patient care and relieve unnecessary health care costs.

## Allied Health

### A49 Qualitative studies of teen experiences living with Food-Induced Anaphylaxis (FIA): a meta-aggregation

#### Sara F. Johnson, Roberta L. Woodgate

##### College of Nursing, Rady Faculty of Health Sciences, University of Manitoba, Winnipeg, MB, Canada

###### **Correspondence:** Sara F. Johnson

*Allergy, Asthma & Clinical Immunology* 2017, **13(Suppl 1)**:A49

**Background:** As FIA prevalence increases and commonly outgrown allergies persist longer, chronic management becomes increasingly important. Exploring teen experiences with FIA offers insight regarding increased rates of fatal anaphylaxis and greater emergency care needs for teens. This meta-aggregation seeks to understand how teens experience living with FIA as a basis for improving health services for this population.

**Methods:** Using the Joanna Briggs’ Institute guidelines for meta-aggregation, a literature search was completed in July 2015 using 5 relevant databases. Both authors undertook critical appraisal using the JBI-QARI tool, with consensus reached by discussion. In studies with mixed populations, we included only data from teens (age 12–19) with FIA. Line-by-line extraction of findings was grouped into subcategories, categories, and syntheses.

**Results:** Ten studies from 2007 to 2015 met inclusion criteria. Most were conducted in Europe, and half contained mixed populations. We developed 3 syntheses to reflect the central experiences of teens with FIA. These include: (1) Defining the allergic self (experiencing allergy, coming to understand allergy, and redefining normal); (2) Finding a balance (the burden of allergy, ways of responding, and moving towards independence); and (3) Controlling the uncontrollable (being vigilant, negotiating risk, and systemic influences). These experiences encompass the importance of allergic identity/understanding and difficulties coping with burdens of FIA, reflecting the complex risk interactions teens must navigate.

**Conclusion:** Future research would benefit from congruent epistemology and methodology, and a wider array of perspectives to capture a more detailed sense of the experiences of teens with FIA. Teens must manage their allergic identity to balance social risk and risk of anaphylaxis when many factors are beyond their control. Public awareness and health policy play roles in the context for teen experiences living with FIA. This understanding helps broaden how we conceptualize the needs of teens living with FIA, informing ongoing care and management.

